# The role of frailty in shaping social contact patterns in Belgium, 2022–2023

**DOI:** 10.1038/s41598-025-96662-8

**Published:** 2025-04-15

**Authors:** Neilshan Loedy, Lisa Hermans, Maikel Bosschaert, Andrea Torneri, Niel Hens

**Affiliations:** 1 Data Science Institute, Hasselt University, Hasselt, Belgium; 2 Centre for Health Economics Research and Modelling Infectious Diseases, Vaccine and Infectious Disease Institute, University of Antwerp, Antwerp, Belgium

**Keywords:** Infectious diseases, Disease transmission, Mathematical modeling, Social contact, Frailty, Older adults, Influenza virus, Statistics

## Abstract

Social contact data are essential for understanding the spread of respiratory infectious diseases and designing effective prevention strategies. However, many studies often overlook the heterogeneity in mixing patterns among older age groups and individual frailty levels, assuming homogeneity across these sub-populations. This shortcoming may undermine non-pharmaceutical interventions by not targeting specific contact behaviours, potentially reducing their effectiveness in controlling disease. To address this gap, we conducted a contact survey in Flanders, Belgium (June 2022–June 2023). We collected data from 5995 participants (overall response rates of 19.34%) who recorded 31,375 contacts with distinct individuals. Within this cohort, 14.50% were classified as frail, and 46.85% were classified as non-frail. On average, participants report 5.48 contacts daily, with a median of 4 contacts (IQR: 2–7). These contacts vary based on participants’ age and frailty levels, influenced by the locations of their interactions. Using the collected data, we reconstructed frailty-dependent contact matrices and developed a contact-based mathematical model that integrates participants’ and contactees’ frailty levels to investigate how frailty levels affect transmission dynamics. Incorporating frailty levels into the mathematical model substantially alters the shape of epidemic curves and peak incidences. Such insights might provide useful insights for informing non-pharmaceutical interventions, indicating the potential benefit of similar data collection in different countries.

**Supplementary Information:**

The online version contains supplementary material available at 10.1038/s41598-025-96662-8.

## Introduction

Individuals within a population exhibit varying characteristics that influence infectious disease transmission^1,2^. These individual-level heterogeneities can arise from various sources, such as varying individual infectiousness or the infectious period (physiological mechanisms) or varying contact rates with infection sources (behavioural mechanisms)^2^. In various environments, individuals’ behavioural heterogeneity can be seen in varying degrees of contact interactions, being dependent on biological ages, genders, health conditions, and social classes (with expected variations in e.g., seasons)^3–5^. These heterogeneities, particularly among older individuals, can significantly contribute to the infectious disease burden (e.g., 92% of hospitalisations for Herpes Zoster Virus in Italy and 86% for SARS-CoV-2 Virus in England, Scotland, and Wales occur in individuals over 50 years of age^6,7^). Therefore, understanding contact patterns within this subpopulation is crucial. This need is further amplified by demographic trends suggesting a growing elderly population, which is more susceptible to infections due to age-related immune decline^8,9^.

Frail and older populations account for a significant proportion of the health burden, influenced by both biological age and individual health conditions^10^. Therefore, understanding transmission dynamics is crucial for adequately determining suitable public health interventions. However, while transmission models offer valuable insights in understanding disease dynamics, their accuracy is dependent on reliable data on social interactions. This reasoning motivates the collection of social contact data, primarily collected through diary-based social contact surveys, which has been instrumental as an essential tool in parameterising mathematical models to understand the dynamics of infectious disease within the population^3,11–14^. An example is the groundbreaking POLYMOD study, which is a large-scale survey that gathered data from eight European countries with age groups focusing mainly on young adults and adults^3^. Another example is the CoMix study, which aimed at collecting social contact behaviour during the COVID-19 pandemic, highlighting the impact of non-pharmaceutical interventions following the outbreak^12–20^. However, there are significant knowledge gaps, as social contact surveys that specifically focus on older adults are scarce. This demographic, despite its substantial contribution to disease burden, has been underrepresented in existing studies, hindering a comprehensive understanding and precise modelling of transmission dynamics within this population.

While studies have examined social contacts that specifically target older individuals^21^, individuals with chronic illnesses^22^, and frail individuals^5^, there is still limited consensus on the most effective survey methods, especially for studying social contacts in these groups. While research suggests that older individuals are often more inclined to prefer paper-based questionnaires^23^, digital surveys may be favoured by research institutions due to faster distribution and cost-effectiveness. However, the feasibility of digital surveys for older populations remains debatable. As the global population ages, collecting reliable data on social interactions in older individuals becomes increasingly important. Identifying the most effective survey methods will help design inclusive studies that inform public health interventions and policies, ultimately improving health outcomes and quality of life for this subpopulation.

This study presents the findings of the Epicurus contact survey study, conducted in Flanders, Belgium. The study explores social mixing patterns across age groups and frailty levels, focusing on older individuals, by including those with chronic conditions and/or residing in healthcare facilities. In the scope of this study, we use the term “frailty”, to indicate a condition that varies between individuals and refers to those at increased risk of poor clinical outcomes, resulting from a decline in multiple physiological systems, hospitalisation, requiring long-term assisted care, or increased mortality^24^. Furthermore, we investigate how mixing patterns and frailty levels of participants and their contacts might influence the spread of respiratory infectious diseases using a mathematical model. We did so by decomposing the age-structured social contact matrices obtained from the survey based on frailty levels, assuming hypothetical levels of assortativity between these groups. In parallel, we assess the most suitable survey modes in this setting to refine data collection protocols and guide the design of future large-scale, multi-country studies across different countries.

## Methods

### Ethics statement

Participants opt-in for the study voluntarily, with informed consent obtained from all participants. The study received approval from the Comité voor Medische Ethiek UHasselt (CME UHasselt) under Reference Number CME2021/110 and was performed in accordance with relevant guidelines and regulations. Analyses were conducted using pseudo-anonymized data. The English translations of the study protocol and survey questionnaire are presented in the Supplementary Materials.

### Study design

Information on social contacts was obtained cross-sectionally, with the assistance of a market research company. The survey employed a randomized design within the general population, conducted from June 2022 to June 2023. The list of participants was obtained from a database of the National Registry acquired from Statbel, and delivered directly to the market research company. The sampling method received approval from the Federal Public Services for Home Affairs under reference number 040/2022. Participants were invited to join the study by mail. The invitation included several survey completion options: participants could fill out the enclosed paper form and return it by mail free of charge, or they could complete the survey online through the Computer-Assisted Web Interface (CAWI) by following the instructions provided in the mail. For participants younger than 18 years old, a parent or legal guardian within their household completed the questionnaire as a proxy. To encompass individuals residing in care facilities, sampling was done through a list of government-recognised facilities. Data collection within these facilities was carried out through in-person interviews (can be either paper-based or CAWI). In this study, we refer to a facility which provides long-term housing and a range of services for individuals aged 65 years and older who can no longer live independently at home. These services include residential assistance, daily assistance, daily task support, and health care, including nursing care. Moreover, a dedicated mobile app was also available for participants aged 21–60. Initially, no incentives were offered to participants who completed the survey using the app. However, due to low participation rates through this mode (0.5%) in the first survey round, we introduced incentives to encourage participation until the target population quota for this method was met. Due to financial constraints, incentives were terminated once the quota was reached, which may have limited further app-based participation.

Five target populations (general population, individuals residing in care facilities, those with chronic conditions, people with ILI (Influenza-Like-Illness), and the general population via app) were recruited using a stratified sampling approach adjusted by age to ensure a representative sample of the Flanders population (Table S1). Survey invitations were influenced by the seasonal activity of respiratory infections like influenza and RSV (Respiratory Syncytial Virus), divided into three waves: the first wave was conducted during the Summer period (June 14, 2022–August 18, 2022, and May 10–22, 2023; 20% send-outs), the second wave was carried out in the Fall period (September 5–December 16, 2022; 40% send outs), and the third wave was conducted in the Winter period (January 23–April 14, 2023; 40% send outs). This percentage resulted from the intention to include more participants who experience ILI or RSV symptoms during the fall and winter seasons. To achieve the targeted participation rate among individuals aged 22–99 with chronic conditions or experiencing ILI symptoms, additional invitations were sent during the fall and winter. These invitations were adjusted by age and mainly sent during winter months to increase the sample size of participants experiencing ILI symptoms. Respondents from these additional invitations who did not have chronic conditions, nor were experiencing ILI symptoms, were categorized into the general population. Sampling in care facilities and the app-based group was terminated upon reaching the desired sample sizes due to cost considerations.

The survey collected demographic information (age, gender, religion, country of birth, educational level, occupation, presence of chronic conditions, area, type, and size of residency) and vaccination status against influenza and COVID-19. Additionally, participants were asked to complete a contact diary, recording all face-to-face interactions on a specific day (defined as in-person conversations consisting of three or more words, with or without skin-to-skin contact, between 5 am the previous day and 5 am on the survey day), with the number of potentially recorded contacts in a day was limited to 30 (vs. 29–90 in other studies^3,25^). Details captured for each contact included gender, age range, location(s), intimacy level, frequency, and duration. Participants’ Frailty Index (FI) was measured based on their responses to EuroQol-5 Dimension (EQ-5D) and Short Form Survey-36 (SF-36) questions^26,27^, evaluated with the accumulation of deficits approach^28–31^. This well-validated FI captures a broad spectrum of medical conditions (e.g., comorbidities, physical function, and mental well-being) and has been widely used in various international studies^32^. As done by Curran et al. (2021), we divided each participant into one of three subgroups based on their frailty score: non-frail (*FI* ≤ 0.08 ), pre-frail (0.08 < *FI* ≤ 0.25), or frail (*FI* > 0.25)^31^. If a participant had over 10 quality of life components that were not answered, their Frailty Index (FI) was considered as ‘missing’ unless their available data clearly showed that they already had a high enough score to be classified as frail. Notably, participants aged 2 years or younger are assumed to be categorized as non-frail as they were not yet able to complete the EQ-5D and SF-36 questions.

### Statistical analysis for the number of reported contacts

We developed right-censored negative binomial (NBI) generalized linear mixed models to examine the factors influencing the average number of contacts reported inside and outside the participant’s home. The reported contacts were right censored at 30 contacts due to a limited number of possible diary entries and were fitted using penalized maximum likelihood within the *‘gamlss’* package in R^33^. The models incorporated frailty levels and adjusted for other participant characteristics. We first applied a random forest analysis to identify the main predictors, after which we modelled the aggregated number of contacts via a generalised linear model assuming a negative binomial distribution for the response variable. Likelihood ratio tests were used to select the model that best fits the data. In this study, the term “home” refers to the domicile of the participant, encompassing houses, apartments, or healthcare facilities. Social mixing patterns were further investigated by constructing age-stratified ([0, 50), [50, 60), [60, 75), …, [90, 100)) contact matrices for different locations of contacts (inside or outside the home)^3^. The *‘socialmixr’* package in R was employed with post-stratification weights to account for the distinction between weekdays and weekends when generating the contact matrices^34^.

### Mathematical compartmental transmission model

In this study, we utilize a discrete-time age-structured compartmental model which incorporates contact matrices for age-specific transmission rates, previously developed by Abrams et al. (2021)^12^. The model is extended to account for age and frailty mixing patterns, enabling the investigation of a COVID-19-like disease’s spread and the assessment of the impact of frailty-dependent interactions within the Belgian population. This involves considering the frailty levels of individuals with whom people at different frailty levels interact. However, empirical data on the level of frailty of contactees is not available, as this information is difficult to obtain due to its personal and subjective nature. To address this, we mathematically decompose the available social contact matrices by assuming that contacts between different frailty levels follow specific assumptions. Let $\:{M}_{y}$ represent a contact matrix, reflecting the average number of contacts reported within a day by individuals falling into a health condition $\:y\:(y\:=\:frail,\:pre-frail,\:and\:non-frail)$ across specific age classes. We decompose contact matrices $\:{M}_{y}$ into $M_{{y,y^{\prime } }}$ where $y^{\prime }$ refers to the frailty level of the contactee (Supplementary Material 1). We assume that the mixing patterns between individuals with frailty levels $\:y$ and $y^{\prime }$ follow one of three scenarios: proportional, uniform, and fully assortative. In the proportional scenario, the assortativity corresponds to the proportion of frailty levels obtained from the survey. In the uniform scenario, assortativity is identical across all frailty levels. Lastly, the full assortativity scenario simulates individuals who only come into contact with others who share the same level of frailty. To isolate the effect of altered contact patterns on disease spread, we assume homogeneous parameters across frailty levels. This simplification enabled us to attribute any observed changes in transmission patterns primarily to variations within the contact matrices.

The proposed model assumes that individuals become infectious (symptomatic or asymptomatic) after a latent period. Symptomatic cases may progress to severe illness, requiring hospitalisations or admission to the intensive care unit (ICU), where they are assumed to be isolated and can not infect other susceptible individuals (Supplementary Material 2). The corresponding 95% confidence intervals presented in the results of this model are obtained using a non-parametric bootstrap method (with 500 replicates) stratified by age and frailty levels, which involves resampling participants with replacement within each specific stratum to preserve the original distribution of these characteristics^35^. We initialized our disease transmission model on March 1st, 2020, to reflect the early stages of the COVID-19 pandemic (Ancestral strain, without vaccination). Simulations were conducted for the Belgian population of 2020 (timestep: 1 day) over a 100-day period. The model uses parameter values from previous studies on COVID-19 vaccination strategies, which were calibrated in parallel with this study (Table S2)^12,36^. To tailor the model to the observed data, we based the initial population on data acquired from Statbel. The age group distribution of individuals exposed to COVID-19 was derived from Willem et al. (2024) and used as the initial values for the exposed compartment. Within this population, individual frailty levels were assumed to be proportional to the susceptibility levels observed in the survey data (Table S3). To compare the impact of different mixing assumptions on transmission dynamics, we conducted two analyses: one using a constant proportionality factor ($\:q$) capturing host- and disease-specific characteristics, and the other using a constant basic reproduction number ($\:{R}_{0}=2.90$) calculated with the next-generation matrix approach for COVID-19 ^12,37^ (Table B1).

## Results

### Study population

Approximately 31,000 letters (55% main and 45% additional invitations) were dispatched to the participants within the random sample generated by the National Registry. The overall response rate was 19.34% ( $\:{n}=5995$), with the highest response rate coming from the general population of aged 50–79 (22%) and 80–89 years old (22%). The majority of subpopulations from the collected sample exceeded the desired target study population, except for the ILI subpopulation, which only reached 3% of the expected quota (Table S1). Notably, a preference for the paper-based survey (66.67%) was observed compared to the computer-based version (CAWI or app-based) (33.32%). Of the initial respondents, 272 individuals did not consent to participate, and one participant was excluded because they did not comply with the contact reporting guidelines (the reported contact was not from the day preceding the survey). The final analysis included 5723 consenting participants (44.18% male, 53.45% female) who reported 31,375 contacts (Table S4).

The mean participant age was 53.4 years, with a median of 59 years (IQR: 37–71). The majority of survey participants are aged 60–69, while around 27% are older than 70 years. Roughly 60% (2200 out of 3725) of the participants who opted to complete the survey on paper are older than 60 years. More than 60% of participants in the 20–29 and 30–39 age groups chose to complete the survey using CAWI or the dedicated app. For participants residing in healthcare facilities, 68% (185 out of 271) chose to complete the survey using the paper format. Overall, 75.3% of participants aged 60–69, 84.5% of those aged 70–79, 81.4% of those aged 80–89, and 79.7% of those aged 90–100 chose the paper-based survey (Table S5). The province of Antwerp had the highest percentage of survey participants (26.5%), while Limburg had the lowest (14.5%). The survey was completed without any help by 75% of the participants. The paper-based survey skewed towards individuals over 60 (Fig. S1). The data indicates that individuals over 70 years old generally tend to engage in volunteer work or remain unemployed (either retired or capable of working but not currently employed). Chronic health conditions were reported by 33.4% of participants, all exceeding 50 years old. Most participants resided in households of size 2 (33.2%), 3 (18.4%) or 4 (16.23%). Only 10.69% of our participants lived alone, while 9.8% of the participants resided in a household of size five or more. A small proportion (3.5%) resided in healthcare care facilities (retirement or nursing homes (ROB: *Rustoord voor Bejaarden*; RVT: *Rust-Verzorgingstehuis*)), especially older individuals. Furthermore, frailty assessment revealed a significant association with biological age, with older participants tend to be in the more frail categories. Among the total sample, there were 830 participants (14.50%) classified as frail, 1,749 (30.56%) as pre-frail, 2,681 (46.85%) as non-frail, and 463 (8.09%) were categorized as missing, as described in Sect. 2.1.

### Contact behavior and mixing patterns

**Fig. 1:**
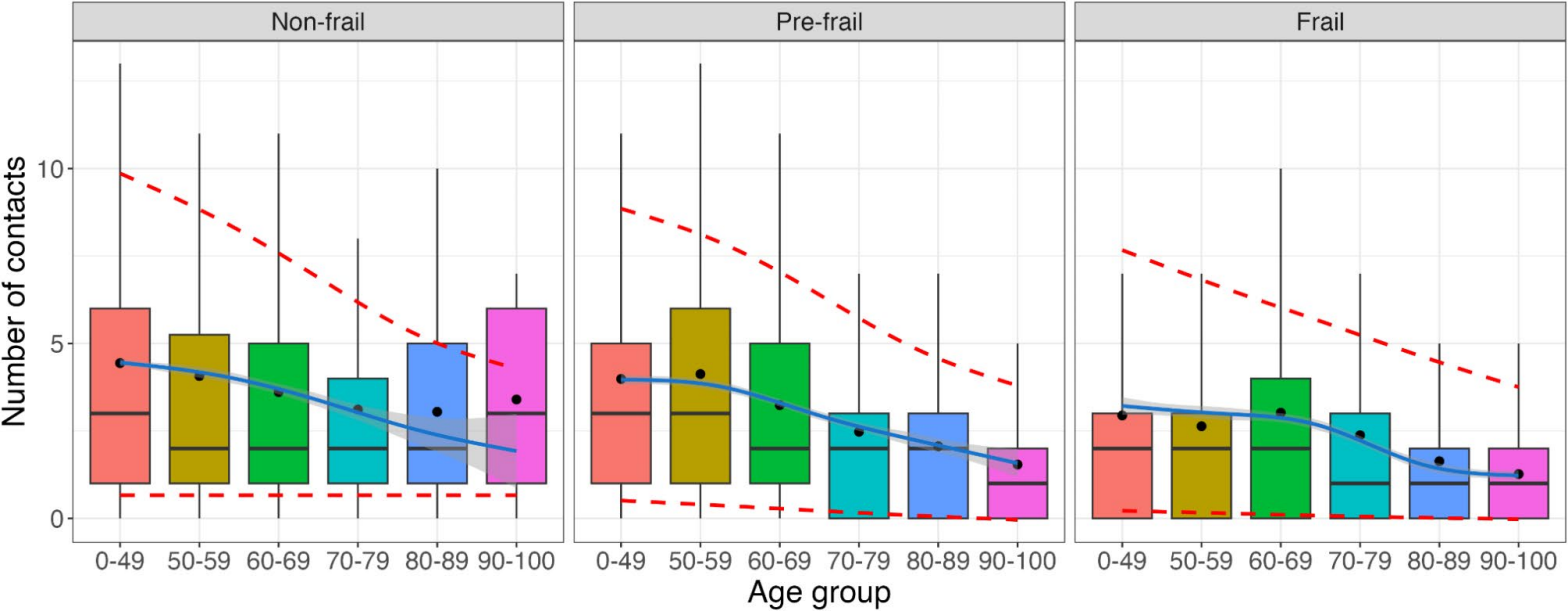
Number of contacts reported outside the home by participants’ age group and frailty level. The plots show the distribution of contacts, the average number of contacts (dots), model results (mean as solid blue lines with 95% confidence intervals as shaded area), and 95% prediction intervals (red dashed lines). Outlying points are excluded from the figure.

On average, participants report 5.48 contacts daily, with a median of 4 contacts (IQR 2–7) (Table S6). Only 0.14% of participants reached the upper limit of 30 reported contacts. When considering contacts reported outside the household (Fig. 1), we observed a notable association between frailty and the number of reported contacts. Frailty status influences the average number of contacts, with frail individuals reporting fewer contacts on average ($\bar{x}$ = 4.70, 95% CI [2–6]) compared to non-frail (6.38 [3–8]) and pre-frail (5.07 [2–7]) individuals. The decrease in contacts varies by frailty and is more pronounced in older age groups, with a 26%, 37%, and 40% reduction in average contacts for non-frail, pre-frail, and frail participants over 70 compared to those under 70. Note that the small number of non-frail individuals in the 80–89 and 90–100 age categories (0.8% and 0.2% of non-frail individuals, respectively) may lead to a noticeable discrepancy between the observed average number of contacts and the model-based number of contacts. In addition, our preliminary analyses (Fig. S2) suggest that individuals in the 90–100 age group tend to have either shorter (< 5 min) or longer ( > = 4 h) contacts, while those in the 0–49 age group have fewer longer ( > = 4 h) contacts or very few short ones ( < 5 min). There are few first-time contacts between these two age groups, but individuals in these groups have reported more daily contacts compared to other age groups.

**Fig. 2:**
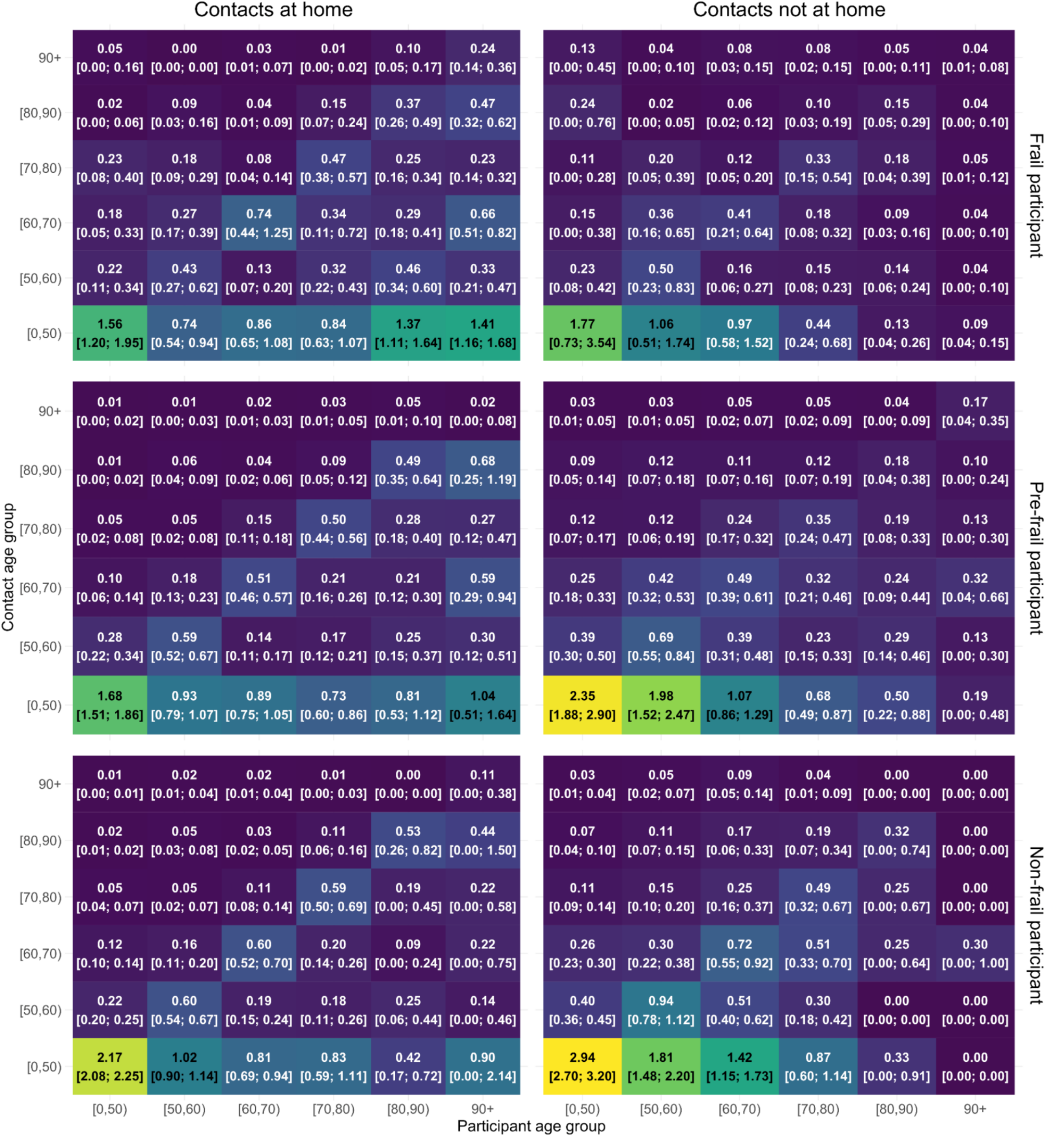
Contact matrices showing the mixing patterns of participants based on their frailty level for contacts reported inside and outside the household, together with 95% confidence intervals obtained from non-parametric bootstrap.

A significant portion of contacts occurs with individuals of similar age, as indicated in the contact matrices for which higher values are reported on the diagonal (Fig. S3). More precisely, older individuals (60–90 years) primarily interact with others in their age range, followed by interactions with younger adults (30–59 years). Intergenerational mixing occurred more inside participants’ homes, particularly between younger individuals (under 18) and adults (30–50 years). Higher contact rates are observed when comparing contacts outside participants’ homes with those reported inside. Pre-frail and non-frail individuals reported more contacts outside their homes compared to frail individuals (Fig. 2). In contrast, frail individuals tended to report more contacts inside their homes. For individuals under 70 with frailty, these trends persisted with a higher number of outside contacts reported compared to those over 70 with frailty, who tended to have more contacts inside their homes. When considering participants with chronic conditions, we observe higher contact rates outside their homes, except for those over 70 years old, who reported more contacts within their homes (Figs. S4–S6).

We also examine the effect of different household sizes on the number of contacts with non-household members reported by the participants (Figs. S7–S9). Frail individuals aged 70 and over living alone reported more contact compared to younger frail individuals. This pattern is not observed for pre-frail and non-frail individuals living alone. The number of contacts at home with non-household members was consistently shown to be high for frail participants, in comparison with contacts outside the home, regardless of their residential type. In contrast, for pre-frail and non-frail participants, these contacts are higher when being made outside their home. When specifically examining frail individuals residing in healthcare facilities, we observed a significant discrepancy in mixing patterns between contacts with household members (individuals sharing the same room within a healthcare facility) and contacts with non-household members (Fig. S10). Frail and pre-frail individuals residing in healthcare facilities exhibited a notably low average number of contacts with household members. On the other hand, the average number of contacts with non-household members was higher, particularly among older individuals (greater than 70 years old for frail individuals and greater than 60 years old for pre-frail individuals). Due to the very small number of non-frail individuals ($\:n$ = 4) residing in healthcare facilities in our study population, we did not include their contact patterns in the figure.

### Contact characteristics

**Fig. 3:**
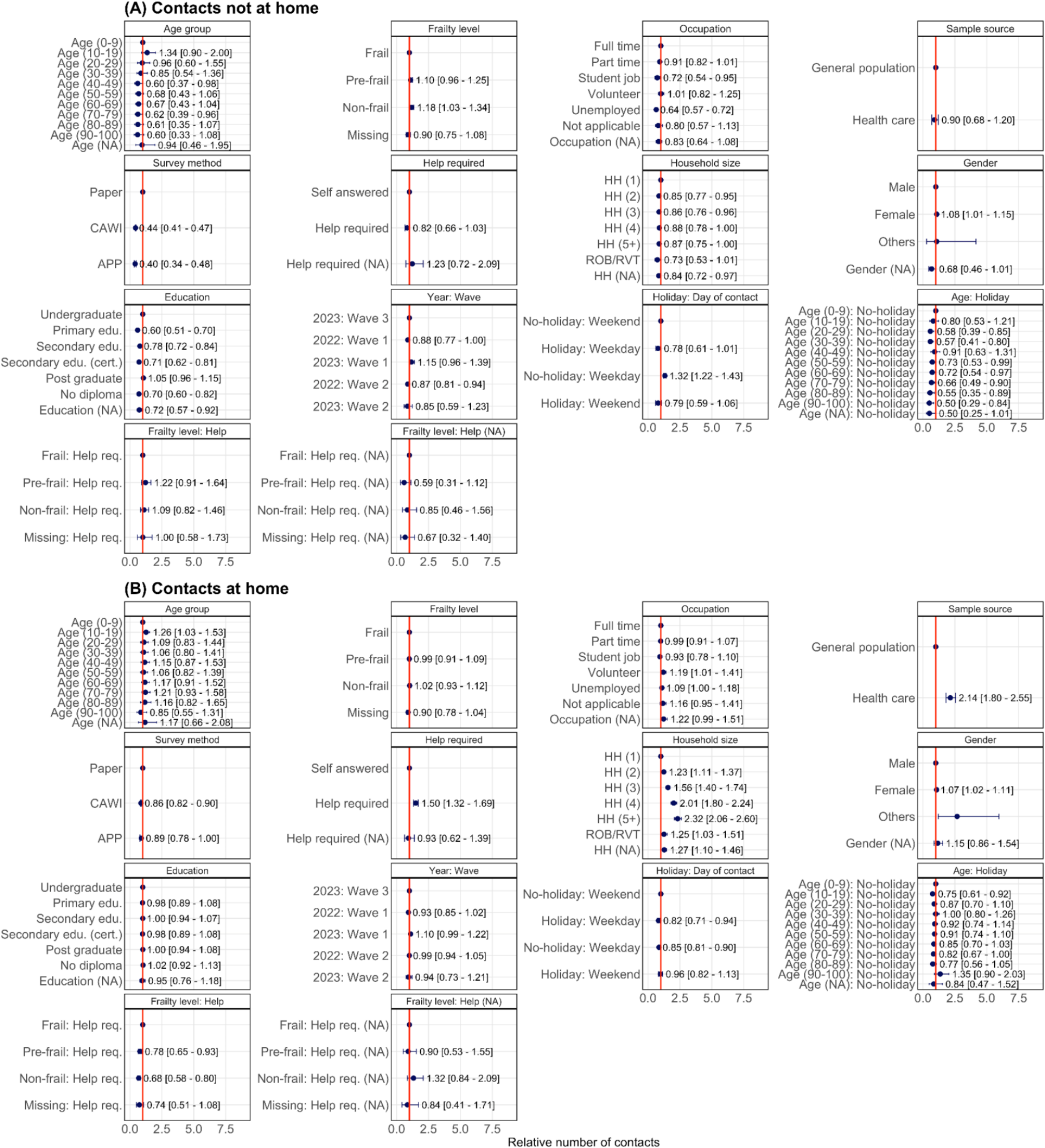
The relative number of contacts (dot) for contacts at home and not at home, along with their 95% confidence intervals based on an NBI regression model. The brackets ”(NA)” indicate that no answer was provided by the participants.

Variables expressing the number of contacts are selected based on the results of both random forest analysis (Fig. S11) and the likelihood ratio test (Table S7). Figure 3 presents the relative incidence (RI) with its 95% confidence interval (CI) obtained from GAMLSS models inferring the number of contacts at home and not at home based on these selected covariates. Some values are not displayed in the figure to maintain readability. The full set of estimates is available in Table S8. While holding other variables constant, the sample drawn from the care facilities reported more contacts (RI: 2.141 [1.797–2.550]) inside the home. The gender variable had a significant effect on the number of contacts reported, with women reporting a higher number of contacts inside (RI: 1.077 [1.012–1.146]) and outside (RI: 1.066 [1.020–1.113]) the home. People who completed the questionnaire on paper tend to report more contacts, with a relative incidence of 2.293 [2.136–2.463] and 1.160 [1.106–1.215] for outside and inside home contacts, respectively. Both models show that household size has different effects on contacts at home and not at home. Participants who live with others reported significantly more contacts at home than participants living alone, and such an increase in the number of reported contacts depends on the household size. More precisely, compared to people living alone, we observe a relative increase ranging from 23% for participants living in households of size two (RI: 1.233 [1.111; 1.367]), to 132% for participants living in households of five or more (RI: 2.315 [2.060; 2.602]). In contrast, participants in larger households generally reported fewer contacts outside the home, with relative reductions ranging from 15% for members of households of size two (RI: 0.854 [0.770; 0.948]), to 13% for members of households with size five or more (RI: 0.866 [0.753; 0.995]) in comparison with those living alone. Unemployed people reported more contacts inside the home (RI = 1.087 [1.001–1.180]) compared to outside the home (RI: 0.641 [0.573–0.716]), and compared to people who worked full time. Lastly, education also has a significant effect on out-of-home contacts, with participants holding undergraduate degrees generally reporting more contacts. More out-of-home contacts were reported during weekdays and non-holidays. There was an interaction between age and holidays on contacts, with older participants (above 50 years old) reporting fewer contacts during non-holidays, compared to children under nine years old. We also observed a statistically significant relationship between frailty levels and the number of contacts reported outside the home, with non-frail individuals reporting significantly more contacts outside the home. This significance was not observed for contacts at home. Lastly, we found an interaction between the number of at-home contacts, frailty level, and whether participants completed the survey with assistance. Specifically, participants who required assistance to complete the survey reported fewer contacts at home.

### Mathematical compartmental transmission model

**Fig. 4:**
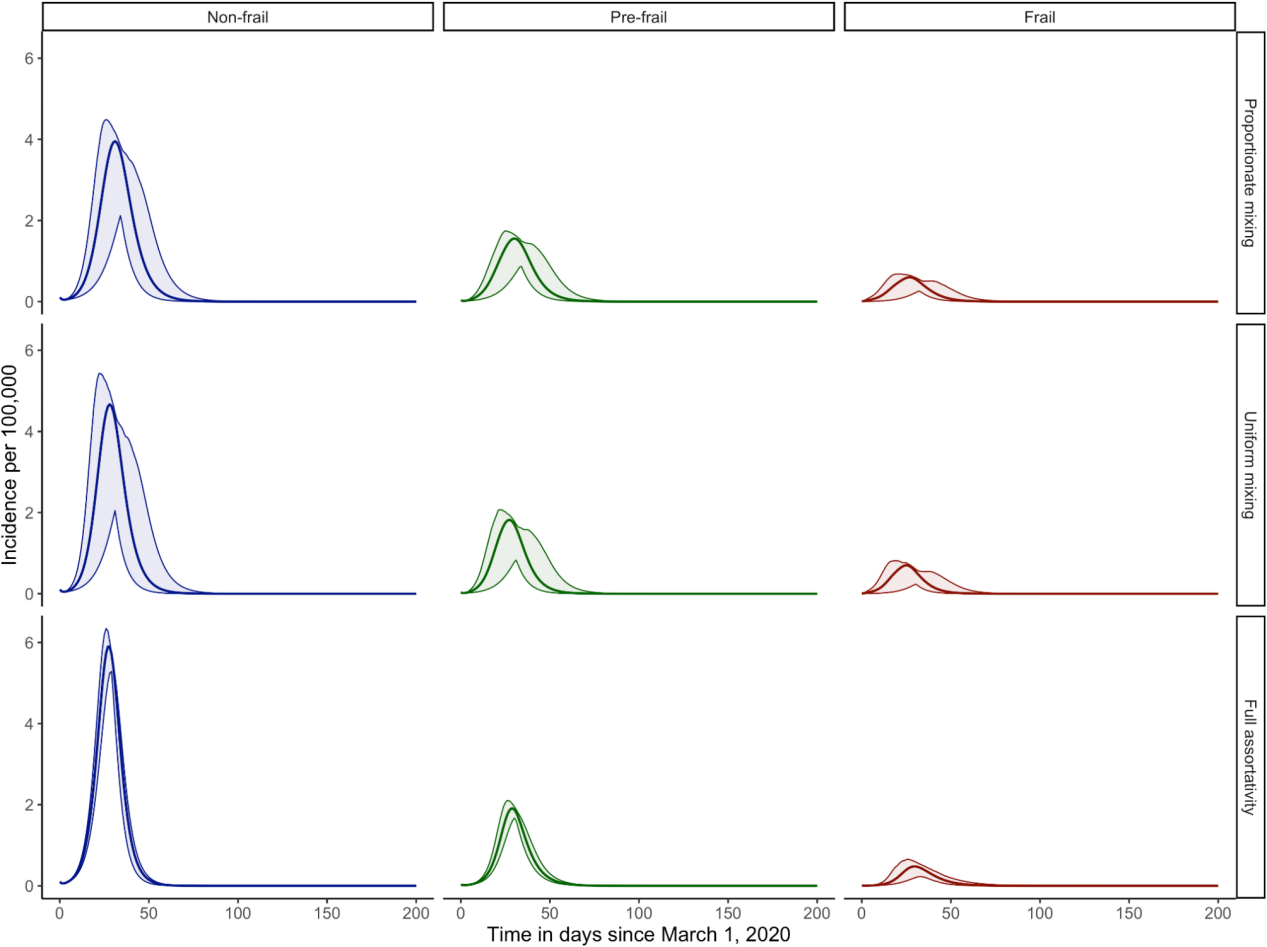
Comparison of COVID-19-like $\:({R}_{0\:}=\:2.90)$ epidemic curves together with 95% confidence intervals obtained from non-parametric bootstrap for the Belgian population with various frailty-based mixing patterns.

The average number of contacts obtained from the decomposition of social contact matrices $\:{M}_{y}$ are presented in Figs. S12–S14 for the assumption of proportionate, uniform, and fully assortative mixing, respectively. The results of the simulation study indicate varying disease dynamics with a constant value of $\:q$ across all mixing patterns (Fig. S15). These variations are driven by changes in reproduction numbers and are further reflected in differences in attack rates – 0.83 [0.80–0.85], 0.86 [0.81–0.87], and 0.58 [0.55–0.62], for proportionate, uniform, and full assortativity mixing, respectively. Full assortativity results in significantly lower epidemic peaks (2.36 [1.82–2.61], 0.46 [0.28–0.52], 0.06 [0.00–0.09] per 100,000 for non-frail, pre-frail, and frail groups, respectively), while uniform mixing yields the highest peak epidemics (5.00 [1.69–5.30], 1.95 [0.63–2.08], 0.74 [0.14–0.81] per 100,000 for non-frail, pre-frail, and frail groups, respectively), compared to other scenarios. When simulating epidemics and keeping a constant value of $\:{R}_{0}$, we observed that mixing patterns among different frailty levels affect transmission dynamics (Fig. 4). While attack rates across the three scenarios—proportional (0.81 [0.77–0.83]), uniform (0.83 [0.79–0.85]), and fully assortative (0.79 [0.78–0.81])—show only a slight difference, the underlying patterns of disease spread vary considerably. Under the full assortativity scenario, non-frail individuals experience the earliest epidemic peak (day 28.00 [26.45–30.00]) and highest incidence rates (5.90 [5.02–6.28] 100,000, respectively). Conversely, frail individuals show the latest peak (day 31.00 [23.00–39.00]) and lowest incidence rate (0.48 [0.20–0.59] per 100,000). Uniform mixing results in the fastest and highest incidence rate for frail individuals (0.70 [0.13–0.76] per 100,000 after 26.00 days [16.00–41.00]), while proportionate mixing shows the lowest incidence rate and latest peak for non-frail individuals (3.96 [1.63–4.18] per 100,000 in 32 [25–42] days).

## Discussion

In this manuscript, we presented the outcomes of the Epicurus study, which was conducted to investigate social mixing behaviors in the Flemish region, Belgium, from June 2022 to June 2023. In particular, our focus was on older individuals, given the established literature demonstrating their significant disease burden^5,8,22^, yet there is still a knowledge gap concerning how their behaviors vary with frailty levels. We conducted simulations using a compartmental model to describe infectious disease spread, incorporating frailty-dependent mixing patterns obtained from the study as a proxy of age-specific transmission rates, and compared outbreak characteristics under various degrees of assortativity. In particular, we have considered the spread dynamics of SARS-CoV-2, given its recent prominence and significant impact. However, it is important to recognize that this model is a simplified representation and epidemiological measures derived from this model should not be used for quantitative assessment of disease outbreaks.

We observed a higher response rate (19.34% vs. 15%) compared to a similar study on older individuals in the Netherlands, which also used national registry sampling^5^. One possible explanation for this difference may be the contrasting study designs, with our study employing a cross-sectional survey and the other utilizing a longitudinal approach^38^. Consistent with Klovning et al. (2009), our study reveals age-related differences in survey mode preferences^23^. The paper-based approach is preferred by older individuals (and especially those living in healthcare facilities), whereas computer-based methods seem to be a preferred mode for younger respondents. There was a marked increase in response rate when incentives were offered (0.5–6%) to fill in the survey with the dedicated app. However, the use of incentives should be considered with caution, as incentives may introduce bias, e.g., by attracting individuals with lower socioeconomic status. Residents of healthcare facilities demonstrated a keen willingness to participate in the study. Though, this may not hold true in other contexts or countries, where factors such as cultural differences, education level, study design, or trust in research may influence engagement levels^39,40^. Recruiting individuals aged 50–75 years from healthcare facilities presented difficulties, as this demographic group typically does not reside in such facilities unless they have specific medical needs. Furthermore, although our sampling strategy considered seasonal fluctuations in respiratory infections, recruiting individuals with ILI symptoms was still difficult, as their willingness to participate may decrease when feeling unwell. Supplementing national registry data with additional information from general practitioners could be of added value, but potential biases towards specific subpopulations should be carefully considered. Nonetheless, the overall age distribution of the study sample met the target quota, suggesting that the sample is representative with limited bias^18^.

Parallel to what was observed by Backer et al. (2023)^5^, our results also show that older individuals with frailty have, on average, fewer social contacts compared to those without frailty. This discrepancy implies that, informing contact-based mathematical models from data collected through social contact surveys from mostly non-frail individuals may therefore introduce bias^22^. Among these contacts, older-frail individuals reported more at-home contacts than not-at-home contacts, which may reduce their exposure to community transmission. This might be attributed to health conditions that limit participation in social activities, especially those requiring higher levels of physical or cognitive functioning^41^. As a result, their social network centers mainly at home, making it their primary social environment. Given that the intimate nature of household interactions often results in higher secondary attack rates (SARs)^42–44^, these results reveal the importance of tailored prevention efforts in households with older and frail individuals. Furthermore, we observed a high number of contacts with non-household members among frail-older individuals residing in healthcare facilities. This pattern may be explained by typical characteristics of these settings, such as shared meals, group activities, and staff rotation^8^. Combined with residents’ frailty, it is pivotal to implement strict measures (e.g., thorough contact tracing, testing, masking, and limited visitation) within this setting to lower the transmission rates during outbreaks of various respiratory infectious diseases^43^. Furthermore, while we briefly explore some contact characteristics in this analysis (Fig. S2), future studies investigating these factors in healthcare facilities would be valuable to best inform mitigation measures in disease dynamics^1^. In addition, we demonstrate how to decompose the contact matrix by incorporating both participant characteristics (e.g., frailty levels) and those of their contacts, while relying on the assumption of assortativity and further emphasize the impact of considering heterogeneity from varying frailty levels within populations on the spread of respiratory infectious diseases, using a contact-based deterministic mathematical model. While maintaining a constant transmission rate across frailty groups, we observed distinct transmission dynamics. This difference arises from changes in the underlying reproduction value when the same $\:q$ value is applied to varying contact rates, signifying the need for caution when extrapolating population-level parameters to specific subgroups.

Our findings suggest that mixing patterns between individuals with varying levels of frailty also play a role in shaping epidemic patterns. Since such interactions are common in real-world settings, understanding assortativity and its impact on transmission dynamics is important. This knowledge can inform the development of more effective strategies to control disease spread. However, it is important to note that these three scenarios simplify reality. We acknowledge this as a limitation and suggest that further research could address this by exploring more detailed assortativity patterns. Future studies could benefit from using close-proximity sensors to collect this data empirically, a method that has already been employed in several confined settings^45^. This minimally invasive approach is particularly important in settings involving older and frail individuals, as it reduces participant budren and enables longitudinal data collection, allowing for the measurement of variation within individuals over time. Moreover, future research should focus on improving model realism by using, e.g., individual based models taking into account healthcare facilities and individual variations in susceptibility (the likelihood of becoming infected with a disease when exposed) and infectivity, one of the key factors influencing transmission dynamics, particularly when considering the different frailty levels within individuals.

This work has several limitations. We examined the relationship between household size, categorized as individuals living alone, those living with others, and residents of healthcare facilities, using separate models (Figs. S16–S18). We observed similar patterns, specifically among individuals living with others. For those living alone, only holidays and sample mode showed a significant impact on the reported number of contacts. The model indicates that frailty, age, and facility size affect the number of reported contacts among individuals in healthcare facilities, though these results should be interpreted cautiously due to limited statistical power and data scarcity within this subpopulation. Further research with larger sample sizes and more comprehensive data from healthcare facilities is needed to improve statistical power and provide more robust estimates of the factors influencing contact patterns. It is important to note that our scope does not encompass comparisons across countries, including any analysis before, during, or after the COVID-19 pandemic, nor does it delve into future projections. Extrapolating these results to other countries may not be wise, as each country possesses unique characteristics (e.g., cultural and educational background, infrastructure, and social structure) that may considerably impact the validity of such extrapolations^46^. However, the insights and methodologies discussed herein can guide potential extensions of this study to a European context.

Our study considers frailty alongside chronic disease, as it offers a broader overview of individual health levels^47^. Nevertheless, we utilized a singular method to calculate frailty, potentially overlooking variation in contact patterns across different frailty measurements. Employing a broader frailty index calculation that encompasses multiple health indicators could provide deeper insights into the interplay between frailty and contact behavior. However, constructing such indices can be cumbersome and may induce participant fatigue if the questionnaire becomes overly lengthy^18^. Future investigations could employ multidimensional frailty scales to unveil a deeper understanding of how frailty influences social interactions. Additionally, while COVID-19 and influenza vaccinations did not significantly impact the number of contacts in this study, exploring the effects of vaccines for other diseases, such as RSV and pneumococcal infections, may provide valuable insights, particularly for the elderly and high-risk groups^48^. Therefore, future research could delve deeper to explore these aspects.

## Conclusion

This study investigated how frailty influences social contact patterns cross-sectionally from a panel focusing on older individuals between June 2022 and June 2023. We found distinct contact patterns across different frailty levels. By integrating these patterns into contact-based mathematical models, we show that accounting for frailty-dependent heterogeneity might impact disease dynamics. These findings demonstrate the importance of considering frailty in infectious disease modelling and suggest the need for further data collection and analysis across broader populations.

## Electronic supplementary material

Below is the link to the electronic supplementary material.

Supplementary Material 1

Supplementary Material 2

Supplementary Material 3

## Data Availability

The data underlying the findings of this study are openly available in Zenodo at 10.5281/zenodo.14810628. To ensure participants’ privacy, the publicly available dataset is released in a more aggregated form than the data directly used in this study. In particular, rather than using detailed information on the ages of participants and contactees, we aggregated them into 10-year age bands. Data on participants’ area of residency was also excluded to further minimize the risk of re-identification. All analysis code is publicly available at https://github.com/neilshanloedy/EpicurusStudy.git.
