# Supplementary material for: The role of frailty in shaping social contact patterns in Belgium, 2022–2023
Source: Sci Rep. 2025 Apr 15;15:12883. doi: 10.1038/s41598-025-96662-8 (PMC12000299; doi:10.1038/s41598-025-96662-8)
Supplement: Supplementary file 1 — Supplementary Material 1 [file 41598_2025_96662_MOESM1_ESM.pdf]

Table 1: Overview of study populations, recruitment methods, survey methods and quota (updated with input Ipsos)

Study population	**GENERAL POPULATION** *excl care facilities*	**RESIDENTS CARE FACILITIES** *excl centres for specific conditions*	**PERSONS WITH CHRONIC CONDITIONS** *(excl care facilities)*	**PERSONS WITH ILI SYMPTOMS**	**GENERAL POPULATION SURVEYED VIA APP** *(excl care facilities)*
**Age**	0-99	50-99	50-99	26-75	21-60
**Applicable recruitment methods**	National registry	List government<br>National registry	National registry	National registry	National registry
**Applicable survey methods**	Paper questionnaire<br>Online questionnaire (on tablet, phone, laptop/pc)	Face to face interview	Paper questionnaire<br>Online questionnaire (on tablet, phone, laptop/pc)	Paper questionnaire<br>Online questionnaire (on tablet, phone, laptop/pc)	App (on tablet and smartphone)
**N persons**<br>**N surveys**	2097<br>2097	270<br>270	170<br>170	250<br>500 (1x when symptomatic; 1x when recovered)	160<br>160
**Quota per age**	Table 2	Table 3	Table 4	Table 5	Table 6
**Gender and geographic distribution**	**Geographic:** representative for Flanders<br>Gender: representative for Flanders, with a lower threshold of 33%				
**Survey timing**	Seasonality:<br>- Week 36 - 52: 40% of respondents<br>- Week 1-18: 40% of respondents<br>- Week 19-35: 20% of respondents				

	(evenly throughout periods)<br>week- vs weekend day: 5/7 vs 2/7<br><br>holiday periods: reasonable distribution				
**Flexibility for subcontractor on sample and age quota**	20% flexibility on N surveys per age (motivation required)			Flexibility to recruit this target population from Study Population 1 instead of adding new subjects (motivation required)	20% flexibility on N surveys per age (motivation required)
