# Supplementary material for: The role of frailty in shaping social contact patterns in Belgium, 2022–2023
Source: Sci Rep. 2025 Apr 15;15:12883. doi: 10.1038/s41598-025-96662-8 (PMC12000299; doi:10.1038/s41598-025-96662-8)
Supplement: Supplementary file 2 — Supplementary Material 2 [file 41598_2025_96662_MOESM2_ESM.pdf]

DSI-GSK Project social contacts - Questionnaire V.5 09/03/2022

Number **Dependencies** **Question** **Code** **Answer**

INFO: In this study, we consider a 'day' or 'past day' to be the most recent 24 hours, starting at 5 a.m. and ending at 5 a.m. the next morning.

Part ³: PERSONAL INFORMATION

* A residential care center offers permanent shelter and care for the elderly, support with daily tasks of care and nursing (=ROB).*
* In a residential care center, places can also be provided for elderly people who are heavily in need of care (=RVT).

Number	Dependencies	Question	Code	Answer
Q1		What is your gender?	1	Male
			2	Female
			3	Other
Q2		What is your date of birth?		DD/MM/YYYY
Q3		Where do you currently live?	5	With parents
			6	Children's health care facility
			7	Adult health care facility (other than ROB, RVT)
			1	House, apartment or service flat
			2	Retirement home (ROB)*
			3	Nursing home (RVT)*
			4	With family (as your adult children or another adult family [illegible]
			5	Other: (open text field)
Q3b		Postal code of your place of residence:		XXXX
Q4a	Q3=1	Do you receive care at home by family or professional caregivers?	1	Yes, family outside household
			2	Yes, family inside household
			3	Yes, professional caregivers
			4	No
Q4b	Q3=2,3,6,7	With how many people do you live here?	1	Less than 10 people
			2	Between 10 and 30 people
			3	Between 30 and 50 people
			4	between 50 and 100 people
			5	More than 100 people
Q4c	Q3 not 2,6,7	Indicate for each person who you live with (or share a room):		
		(1) age		XX
		(2) gender	1	Male
			2	Female

			3	Other
		(3) If they were around anytime during this day	1	Yes
			2	No

Q5		What is the highest level of formal education?	1	I have no formal qualifications
			2	Grade of primary school
			3	Grade of secondary school until second cycle
			4	Grade of secondary school until (three cycles)
			5	A professional of academic bachelor degree
			6	A master or PhD grade

Q6	age>16	Are you currently practicing a profession?	1	Yes, full time (paid work)
			2	Yes, part time (paid work)
			3	Yes, voluntary (1 or 2 days a week)
			4	Yes, voluntary (more than 3 days a week)
			6	Yes, student job
			5	No

Q7	Age>60	Looking back over the entire period between the ages of 20 and 60, which of the descriptions below best describes your past professional life? Please tick the appropriate boxes. (multiple answers possible)	1	Independent
	18<age<60	Which of the descriptions below is most applicable to your professional life? Please tick the appropriate boxes. (multiple answers possible)	1a	Craftsman, trader without employees
			1b	Craftsman, trader with 5 employees or less
			1c	Company manager, trader with 6 employees or more
			1d	Liberal profession
			2	Clerk
			2a	Member of the general management, senior management
			2b	Middle management, not part of the general management
			2c	Other servants
			3	Worker
			3a	Worker with vocational training
			3b	Worker without vocational training
			4	Others
			4a	Housewife / houseman
			4b	Disabled
			4c	Unemployed
			4d	Rentier
	18<age<60		5	Student

Q8b		What is your religion right now?	1	Anglican
			2	Islamic
			3	Jewish

			4	Catholic
			5	orthodox
			6	protestants-evangelisch
			7	vrijzinng
			8	zeg ik liever niet

Q8		In which country were you born?	1	Belgium
			2	Bulgaria
			3	The Netherlands
			4	France
			5	Italy
			6	Poland
			7	Portugal
			8	Spain
			9	Morocco
			10	Romania
			11	Turkey
			98	other country (open field)
			99	I'd rather not say

Q9a		In which country was your mother born?	1	Belgium
			2	Bulgaria
			3	The Netherlands
			4	France
			5	Italy
			6	Poland
			7	Portugal
			8	Spain
			9	Morocco
			10	Romania
			11	Turkey
			98	other country (open field)
			99	I'd rather not say

Q9b		In which country was you father born?	1	Belgium
			2	Bulgaria
			3	The Netherlands
			4	France
			5	Italy
			6	Poland
			7	Portugal
			8	Spain
			9	Morocco

			10	Romania
			11	Turkey
			98	other country (open field)
			99	I'd rather not say

Q9c-d-e-f		In which country was you grandfather/grandmother from father/mother side born?	1	Belgium
			2	Bulgaria
			3	The Netherlands
			4	France
			5	Italy
			6	Poland
			7	Portugal
			8	Spain
			9	Morocco
			10	Romania
			11	Turkey
			98	other country (open field)
			99	I'd rather not say
			999	I don't know

Q10		Did you ever had to deal with a serious disease or medical condition (including mental health, medical injury, disability)?		Yes, myself
				Yes, family
				Yes, because I took care of others
				No

Q10b		Have you ever been diagnosed with any of these respiratory illnesses?		COVID-19
				Pneumonia
				Bronchitis
				Asthma
				Other, namely: (open field)
				No
				I'd rather not say

Q11	Leeftijd>5	Did you receive a COVID-19 vaccine?	1	Yes, 1 dose
			2	Yes, 2 doses
			3	No, not yet
			4	No, I don't want to
			5	I prefer not to say

Q11b		Were you ever diagnosed with COVID-19?	1	Yes
			2	No
			3	I prefer not to say

**CHILDREN**				
Q12		Do you have children?	1	Yes
			2	No
			3	Not applicable
Q12b	Q12=1	How many children do you have? (note: children that are still alive)		
		How old is your youngest child?		
		How often do you see one or more of you children?	1	Daily
			2	Several times a week
			3	several times a month
			4	Once a month
			5	Several times a year
			6	Once a year
			7	Less than once a year

**GRANDCHILDREN**				
Q13		Do you have grandchildren?	1	Yes
			2	No
			3	Not applicable
Q13b	Q13=1	How many grandchildren do you have? (note: grandchildren that are still alive)		
		How old is your youngest grandchild?		
		How often do you see one or more of you grandchildren?	1	Daily
			2	Several times a week
			3	several times a month
			4	Once a month
			5	Several times a year
			6	Once a year
			7	Less than once a year

Q14		Do you smoke or have you smoked (cigarettes, cigars, pipe or e-cigarettes)?	1	Yes, I smoke
			2	Yes, I used to be a smoker, I quit smoking before I was 30
			3	Yes, I used to be a smoker, I quit smoking when I was [illegible]
			4	Yes, I used to be a smoker, I quit smoking after I was 50
			5	No, I never smoked

Q15		Do you drink alcohol?	1	Yes, especially beer
			2	Yes, especially wine
			3	Yes, beer and wine
			4	Yes, especially liquor (for example gin, port, whiskey)
			5	No

Q15b	Q15=1,2,3,4	If so, what is most applicable to you at the moment?	1	I drink 1 or 2 glasses alcohol a day
			2	I drink more than 2 glasses alcohol a day
			3	I drink 1 or 2 glasses alcohol a few times a week

			4	I drink more than 2 glasses alcohol a few times a week
			5	I drink alcohol several times a month
			6	I drink alcohol several times a year

Part 2: HEALTH STATUS

EQ5D + SF36

v1		Do you currently suffer from one of these conditions? (multiple answer possible)	1	Cancer
			2	Diabetes Mellitus
			3	High Blood Pressure
			4	Heart attack/coronary heart disease
			5	Congestive Heart Failure
			6	Cerebrovascular Disease
			7	Arthritis
			8	Chronic Lung Disease
			9	Stomach or Intestinal Ulcers
			10	Migraine
			11	Cataract
			12	Glaucoma
			13	None of the above

v2		Do you have any of the following symptoms?	0	No symptoms
			1	Fever
			2	Chills
			3	Runny or blocked nose
			4	Sneezing
			5	Sore throat
			6	Cough
			7	Shortness of breath
			8	Headache
			9	Muscle/joint pain
			10	Chest pain
			11	Feeling tired or exhausted (malaise)
			12	Loss of appetite
			13	Coloured sputum/phlegm
			14	Watery, bloodshot eyes
			15	Nausea
			16	Vomiting
			17	Diarrhoea
			18	Stomach ache
			21	Loss of taste
			22	Nose bleed

			23	Loss of smell
			24	Confusion
			19	Other: (free text)
			20	Rash

Part 3: DIARY OF CONTACTS ON THE ALLOCATED DAY

Please list all contacts with persons with whom you have been in direct contact and who you have met on this day.

Definition contact: that you have spoken to someone in his / her presence less than three meters away (telephone contact or contact via [illegible]

OR

that you have had a physical contact with someone: if you have touched someone (even if this was without having a [illegible]

Other instructions A 'day' starts at 5 AM and ends the next day at 5 AM

Chronological

Definition household Members of your household are people you live with on a daily basis. (for example, co-residents in a rest home count here)

Provide for each contact

Q16

Can you first indicate which day is the past day? By this we mean the day that started yesterday at 5 a.m. and ended at [illegible]		Date

Age		XX

Gender	1	Male
	2	Female
	3	Other

He/she is	1	A household member
	2	A family member
	3	A caregiver
	4	A friend
	5	Other: (open text field)

Contact locations (multiple answer possible)	1	At home (nursing homes, retirement homes etc. included)
	2	Work
	3	On the way (car, train, bake, on foot)
	4	With family
	5	Other: (open text field)
	6	At school
	7	Hobby or sports association

Did you have this contact inside or outside?	1	Indoor
	2	Outdoor

Did you had physical contacts (shake hands or give a kiss)?		1	Yes
		2	No

How often do you see this person?		1	Almost every day
		2	Several times a week
		3	Several times a month
		4	Several times a year or less
		5	First time

Time spent with this person during the whole day:		1	Less than 5 minutes
		2	Between 5 and 15 minutes
		3	Between 15 minutes and 1 hour
		4	Between 1 and 4 hours
		5	Longer than 4 hours

Did this person use/wear a mounth mask?		1	Yes, the hole time
		2	Yes, part of the time
		3	No

Part 4: TIME SPENT ON THIS DAY

Q17

Mark for each time block
(1) place where you spent most of your time
(2) how far from home you were

(create table)

Time block
05:00-08:00
08:00-10:00
10:00-12:00
12:00-14:00
14:00-16:00
16;00-18:00
18:00-20:00
20:00-22:00
22:00-24:00
24:00-02:00
02:00-05:00

Place where you spent most of your time
At home (nursing homes, retirement homes etc. included)
On the way (car, train,bike)
At work

With family
At school
Hobby or sports association
Other

Distance from home
0 - 1 km
2 - 9 km
10 - 25 km
26 - 75 km
76 km or more

Part 5: A FEW MORE QUESTIONS ABOUT THIS DAY

Q18		Was this a regular day for you?	1	Yes
			2	No

Q18b	Q18=2	Why was it not a regular day?	1	I was temporarily sick
			2	I was on holiday
			3	I was in quarantine
			3	I took care of a family member that is temporarily ill
			4	Other: (open text field)

Q19		How many contacts did you likely not report? Perhaps you forgot some or there were too much.	1	None
			2	1 to 4
			3	5 to 9
			4	10 or more

Q20		Did you use public transportation this assigned day? (If yes, multiple answers possible)	1	Yes, tram or bus
			2	Yes, train
			3	Yes, other: (open text field)
			4	No

EXTRA

Q21		Were you able to complete the questionnaire completely independently or did you receive help from another person?	1	I have filled in/answered it completely myself.
			2	I have received help from a family member, friend or other confident.
			3	I discussed my participation with a family member, friend or other conident.
			4	I asked for help/more information using the studies contact [illegible]

Q21b		Please indicate with a score between 0 and 10 how difficult (=0) or easy (=10) you found completing/answering the [illegible]		Open field
Q21c		Do you have other comments about the study or do you see areas where we can improve the approach of the study or questionnaire in the future?		Open field
