# Supplementary material for: The role of frailty in shaping social contact patterns in Belgium, 2022–2023
Source: Sci Rep. 2025 Apr 15;15:12883. doi: 10.1038/s41598-025-96662-8 (PMC12000299; doi:10.1038/s41598-025-96662-8)
Supplement: Supplementary file 3 — Supplementary Material 3 [file 41598_2025_96662_MOESM3_ESM.pdf]

Supplementary Material for: "The Role of Frailty in Shaping Social Contact Patterns in Belgium, 2022-2023"

Neilshan Loedy*[1], Lisa Hermans[1], Maikel Bosschaert[1], Andrea Torneri[1], Niel Hens[1,2]

[1] Data Science Institute, Hasselt University, Hasselt, Belgium
[2] Centre for Health Economics Research and Modelling Infectious Diseases, Vaccine & Infectious Disease Institute, University of Antwerp, Antwerp, Belgium

*Corresponding author: neilshan.loedy@uhasselt.be

Table of Contents:

1. Decomposition of social contact matrices **1**
2. Mathematical compartmental transmission model **2**
3. Supplementary materials (Figures) **4**
4. Supplementary materials (Tables) **8**

1. Decomposition of social contact matrices

To explore the impact of behavioural changes following the frailty status of individuals, we derive social contact matrices to reflect mixing patterns between people with different frailty statuses. The social contact matrix, denoted as M, represents the average number of daily contacts reported by individuals within specific age classes. Here, each element m_{ij} depicts the mean number of contacts reported by participants in age class i with individuals in age class j. In this study, we define three distinct matrices, namely M_{frail}, $M_{pre-frail}$ and $M_{non-frail}$, delineating mixing patterns associated with individuals classified as frail, pre-frail and non-frail with the general population, respectively. Let W_y be a matrix representing the proportion of individuals falling into a health condition y (y = frail, pre-frail, non-frail), defined as

$$W_y = \frac{w_{y,i\cdot}}{\sum_{a=1}^{i} w_{y,a}}.$$

With $w_{y,i\cdot}$ the total number of individuals with a health condition y in age class i and $\sum_{a=1}^{i} w_{y,a\cdot}$ is the total number of individuals in a health condition y. Based on the law of total expectation, the conditional expected number of contacts made by individuals can be written as:

$$E(X = M) = \sum_{y=1}^{3} E(X = M \mid Y = y) \cdot W(Y = y).$$

It is important to note that we can decompose the mixing patterns further, by looking at the fact that individuals with a frailty level y can interact with others in any of the frailty categories (e.g., $M_{frail,\,non-frail}$ represents contacts between frail and non-frail individuals). As such, contacts made by individuals in a frailty condition y can be written as:

$$M_y = \sum_{y'=1}^{3} M_{y,y'}.$$

Let $\xi_{y'} \in \mathbb{R}$, such that $0 \le \xi_{y'} \le 1$ be the degree of assortativity between individuals with health condition y and another individual with health condition y', with $\sum_{y'=1}^{3} \xi_{y'} = 1$. The degree assortativity utilised in this analysis can be seen in Table A1. Let $\rho_{i-y,\,j-y'}$ is the matrix of probabilities with each element representing the probability of an individual with the frailty status y in age class i making contact with an individual in age class j with frailty status of y'. This can be defined as:

$$\rho_{i-y,\,j-y'} = \frac{w_{y',i\cdot}\,\xi_{y'}}{\sum_{y'=1}^{3} w_{y',i\cdot}\xi_{y'}}.$$

with $w_{y',i.}$ the total number of individuals with a health condition y' in age class i. Hence, contact matrices that reflect mixing patterns for individuals in the frail status y can be written as:

$$M_{y,\,y'} = M_y \times \rho_{i-y,\,j-y'}.$$

Furthermore, the per capita contact rates for participants with the frailty status y of age i with individuals of age j with frailty status of y' can be denoted by $c_{i-y,j-y'}$. This matrix is linked to the social contact matrix by [1],

$$c_{i-y,j-y'} = \frac{M_{y,y'}}{N_{j-y'}}$$

With $N_{j-y'}$ corresponds to the population size in age class j with frailty level y' **(Table S3)**.

Table A1. Degree assortativity among individuals. The proportional degree assortativity values differ slightly from those presented in the main manuscript, as participants with 'Missing' frailty levels are excluded from this analysis.

	**Degree assortativity**	**Value**		
Proportional	ξ_{frail}	0.156		
	$\xi_{pre-frail}$	0.332		
	$\xi_{non-frail}$	0.511		
Uniform	ξ_{frail}	0.333		
	$\xi_{pre-frail}$	0.333		
	$\xi_{non-frail}$	0.333		
		Contacts only with frail individuals	,Contacts only with pre-frail individuals	Contacts only with non-frail individuals
Full assortativity	ξ_{frail}	1	0	0
	$\xi_{pre-frail}$	0	1	0
	$\xi_{non-frail}$	0	0	1

2. Mathematical compartmental transmission model

The following set of ordinary differential equations describes the flows in the proposed age-structured compartmental model:

$$
\begin{aligned}
\frac{d\mathbf{S_y(t)}}{dt} &= -\mathbf{S_y(t)}\lambda_\mathbf{y}(\mathbf{t}) \\
\frac{d\mathbf{E_y(t)}}{dt} &= \lambda_\mathbf{y}(\mathbf{t})\mathbf{S_y(t)} - \gamma\mathbf{E_y(t)} \\
\frac{d\mathbf{I_{presym,y}(t)}}{dt} &= \gamma\mathbf{E_y(t)} - \theta\mathbf{I_{presym,y}(t)} \\
\frac{d\mathbf{I_{asym,y}(t)}}{dt} &= \theta p\mathbf{I_{presym,y}(t)} - \delta_1\mathbf{I_{asym,y}(t)} \\
\frac{d\mathbf{I_{mild,y}(t)}}{dt} &= \theta(1-p)\mathbf{I_{presym,y}(t)} - \{\psi + \delta_2\}\mathbf{I_{mild,y}(t)} \\
\frac{d\mathbf{I_{sev,y}(t)}}{dt} &= \psi\mathbf{I_{mild,y}(t)} - \omega\mathbf{I_{sev,y}(t)} \\
\frac{d\mathbf{I_{hosp,y}(t)}}{dt} &= \phi_1\omega\mathbf{I_{sev,y}(t)} - \{\delta_3 + \tau_1\}\mathbf{I_{hosp,y}(t)} \\
\frac{d\mathbf{I_{icu,y}(t)}}{dt} &= (1-\phi_1)\omega\mathbf{I_{sev,y}(t)} - \{\delta_4 + \tau_2\}\mathbf{I_{icu,y}(t)} \\
\frac{d\mathbf{D_y(t)}}{dt} &= \tau_1\mathbf{I_{hosp,y}(t)} + \tau_2\mathbf{I_{icu,y}(t)} \\
\frac{d\mathbf{R_y(t)}}{dt} &= \delta_1\mathbf{I_{asym,y}(t)} + \delta_2\mathbf{I_{mild,y}(t)} + \delta_3\mathbf{I_{hosp,y}(t)} + \delta_4\mathbf{I_{icu,y}(t)}
\end{aligned}
$$

We initialised our disease transmission model on March 1st, 2020, reflecting the early stages of the COVID-19 pandemic when no vaccines were yet available and only the original strain of the virus was circulating. Let $y = 1, 2, 3$ be the frailty status representing frail, pre-frail, and non-frail individuals and age-specific force of infection in age group $k = 1, 2, ..., K$ with frailty status y is denoted by $\lambda_y(k, t)$. This force of infection represents the instantaneous rate at which a susceptible person in age group k with frailty status y acquires infection at time t. As such, the force of infection is defined as

$$\lambda_y(k, t) = \sum_{y'=1}^{y} \sum_{k'=1}^{k} \beta_{y,y'}(k, k')I_{y'}(k', t).$$

Let $c_{y,y'}(k,\ k')$ are the per capita rates at which an individual with frailty status y in age group k makes contact with an individual with frailty status y' in age group k', per unit of time, and q is a proportionality factor capturing contextual and host- and disease-specific characteristics such as susceptibility and infectiousness. Relying on the so-called social contact hypothesis, we have

$$\beta_{y,y'}(k, k') = q \cdot c_{y,y'}(k, k'),$$

which can be defined as the transmission rates for individuals in an age group k that make contact with an individual in an age group k', per unit of time and $I_{y'}(k', t)$ denotes the total number of infectious individuals in an age group k' with frailty status y' at time t.

Table B1. The proportionality factor for the various scenarios required to achieve a reproduction number of COVID-19-like illness (R_0 = 2.90). Scenario 1 refers to an equal value of q, and Scenario 2 refers to an equal value of R_0.

	Variables	Values
Scenario 1 (proportionate, uniform, and full assortative mixing)	q	0.051
Scenario 2 (proportionate mixing)	q	0.048
Scenario 2 (uniform mixing)	q	0.049
Scenario 2 (full assortative mixing)	q_{frail}	0.106
	$q_{pre-frail}$	0.093
	$q_{non-frail}$	0.081

3. Supplementary materials (Figures)

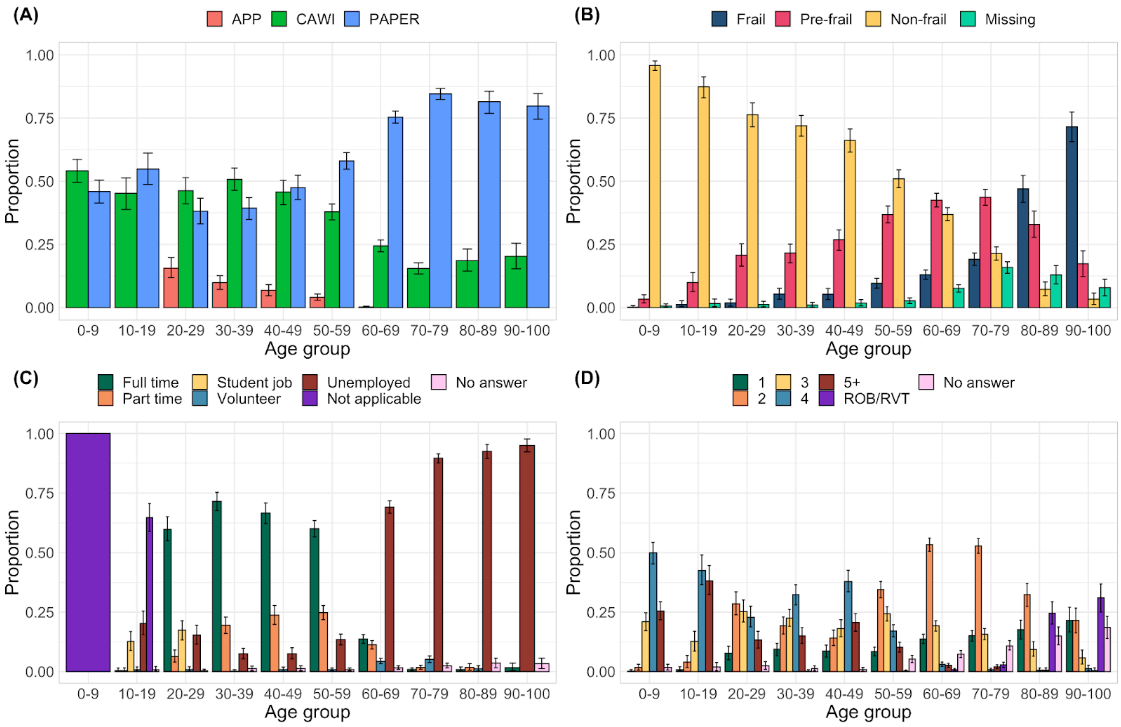

Figure S1: Participant characteristics of the study population. **(A)** Fractions of participants with their corresponding survey methods by age group **(B)** Fractions of participants by frailty level **(C)** Fraction of participants by occupancy **(D)** Fraction of participants with household size.

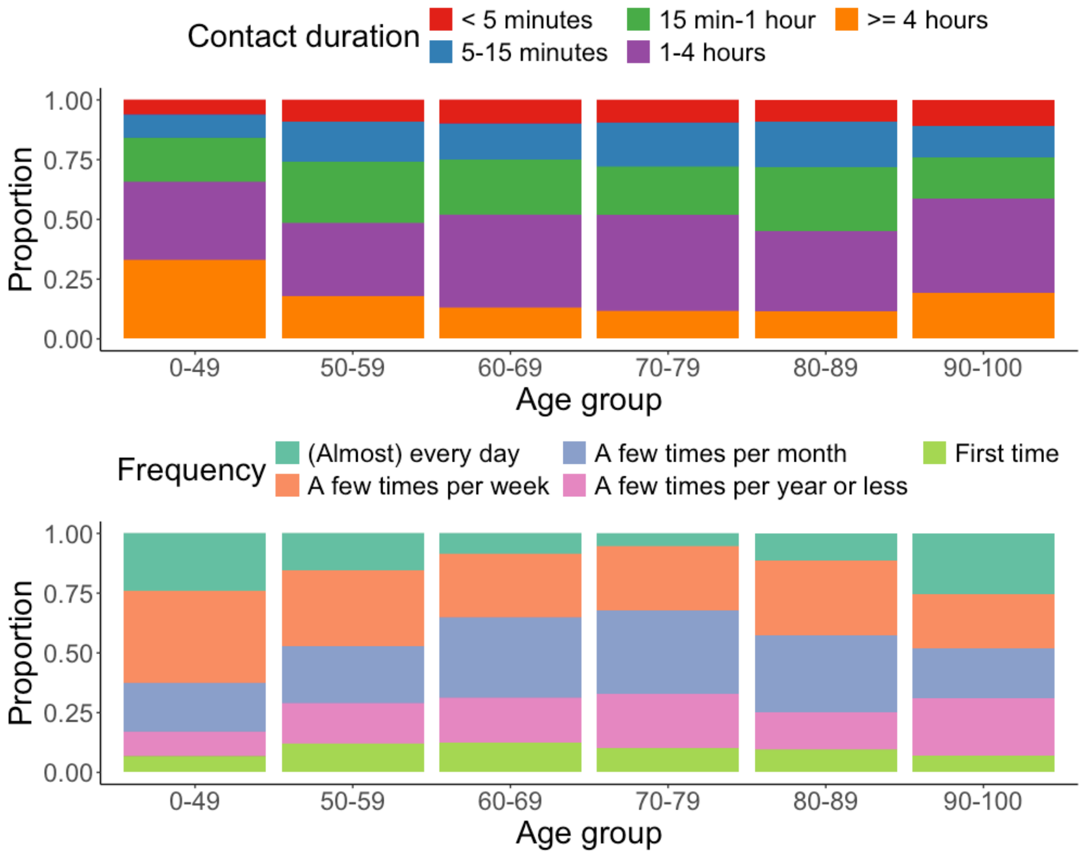

Figure S2. The distribution of (A) contact duration and (B) contact frequency reported outside the home by age group.

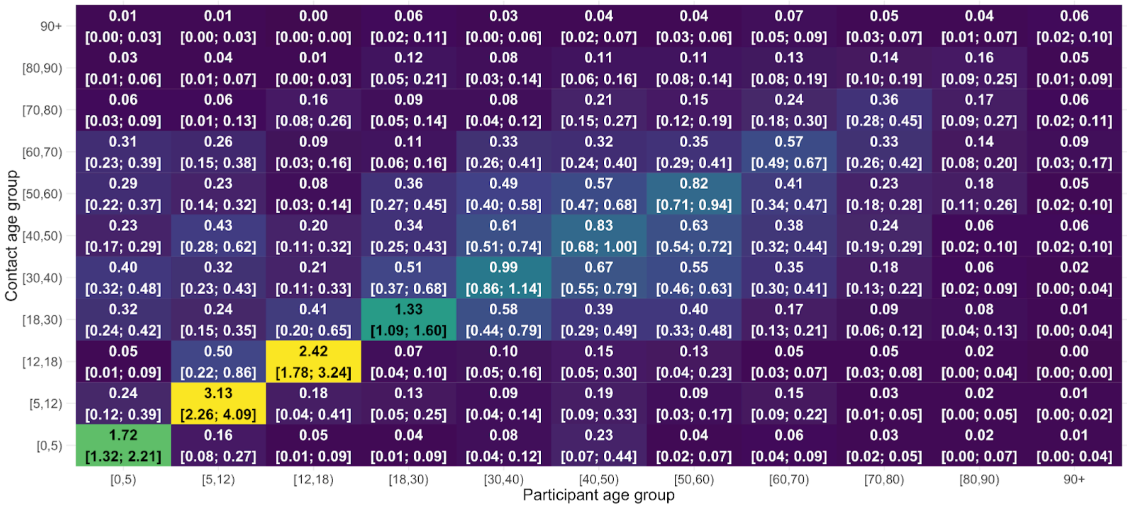

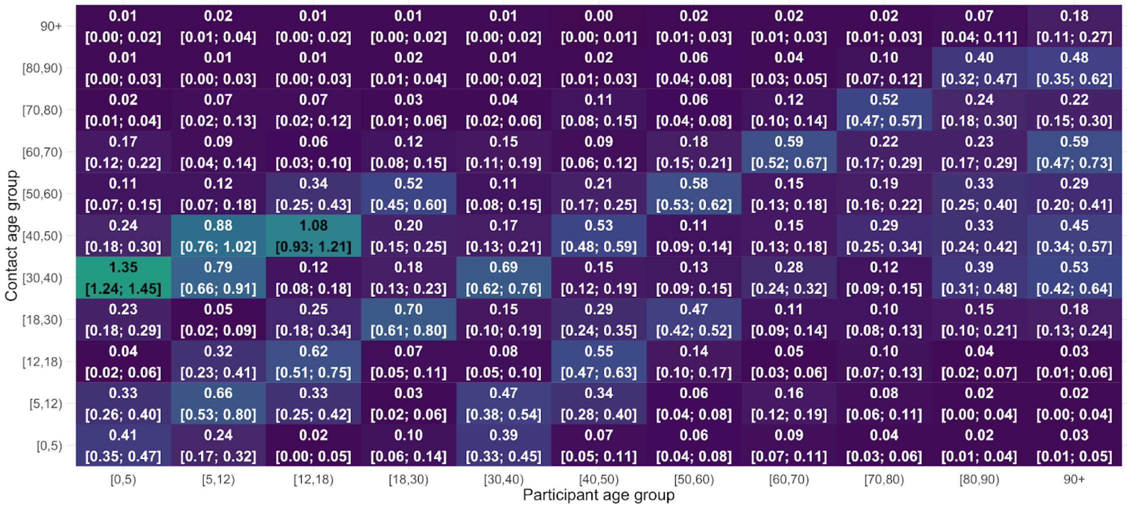

Figure S3: Social contact matrices informing the average number of reported contacts *(Top)* not at home *(Bottom)* at home.

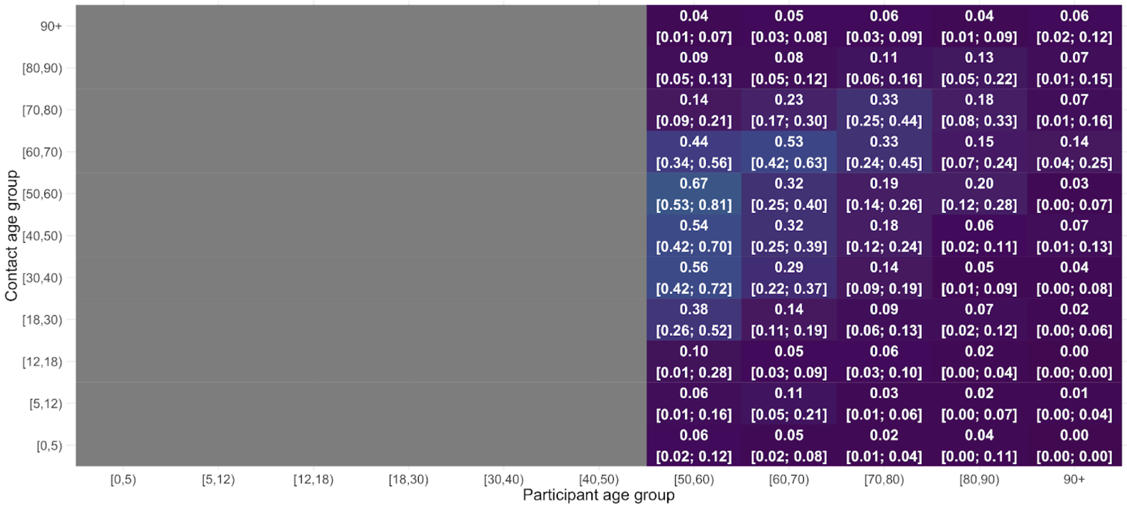

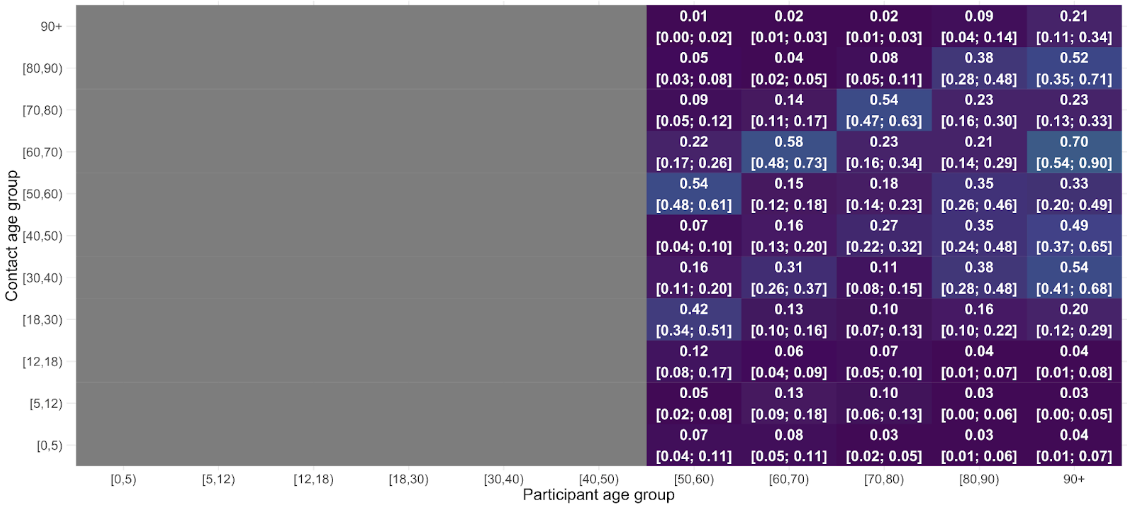

Figure S4: Social contact matrices for chronic participants representing contacts *(Top)* not at home, and *(Bottom)* at home. Note that only participants older than 50 years old reported having a chronic condition.

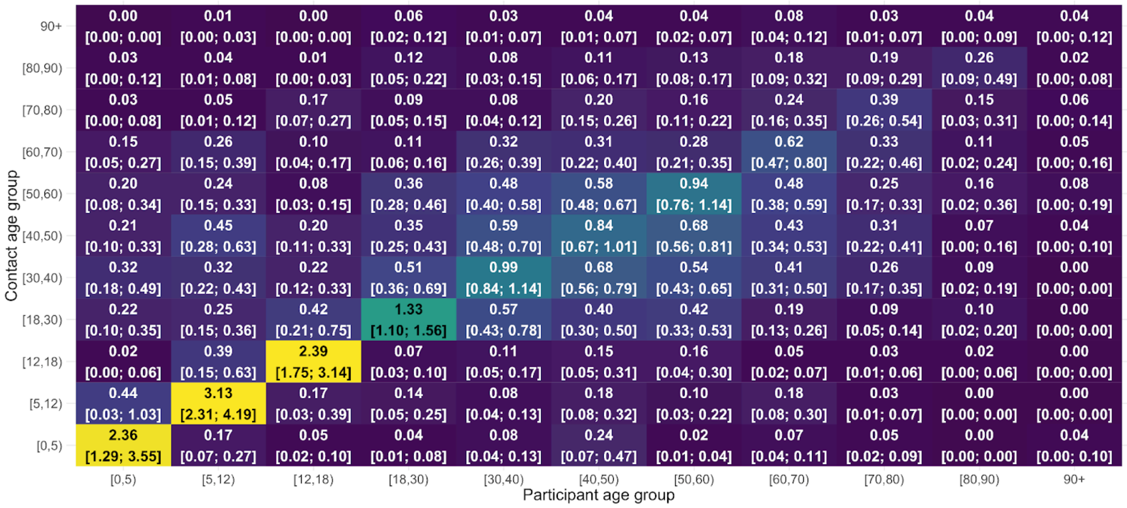

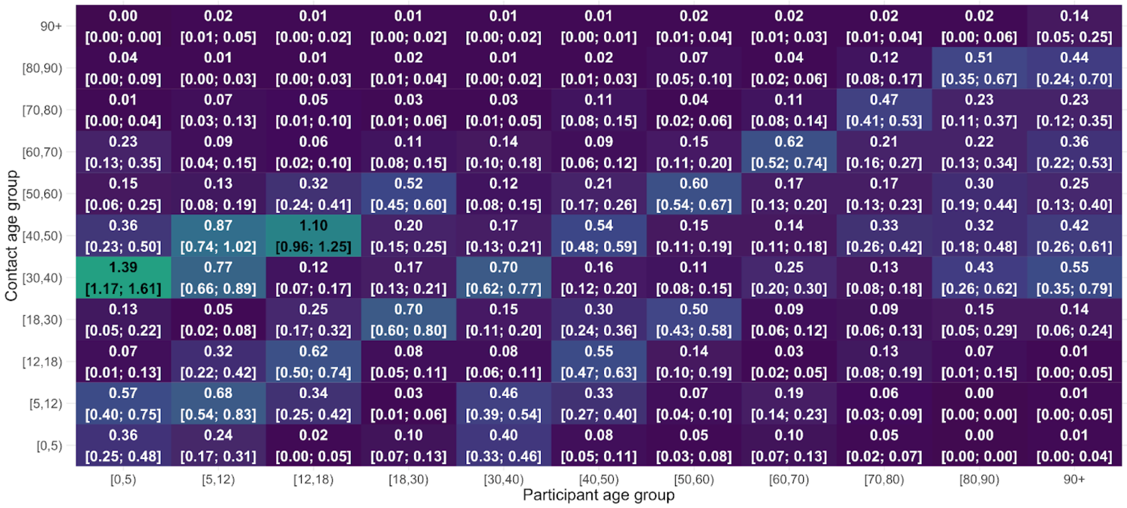

Figure S5: Social contact matrices for non-chronic participants representing contacts *(Top)* not at home, and *(Bottom)* at home.

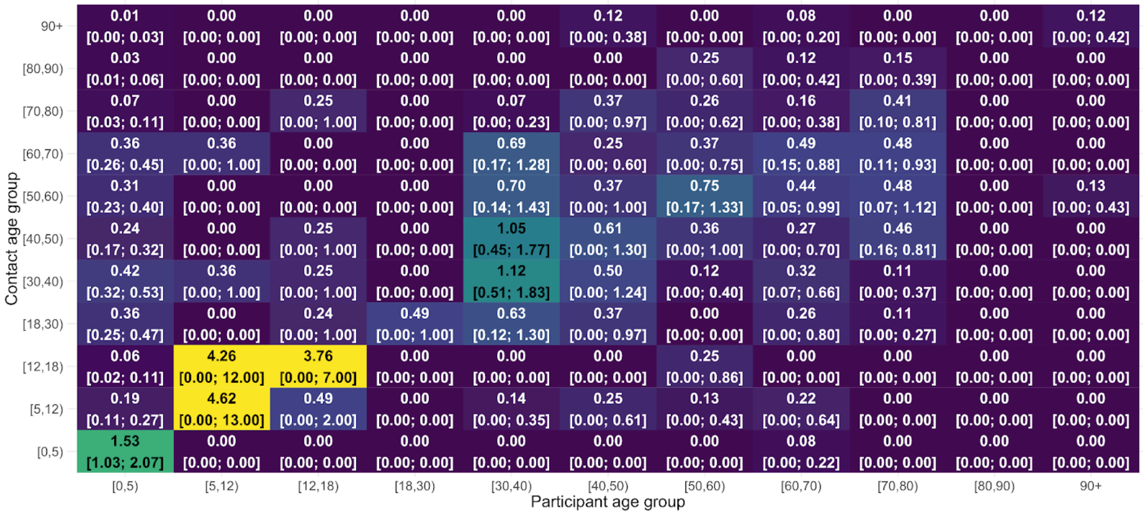

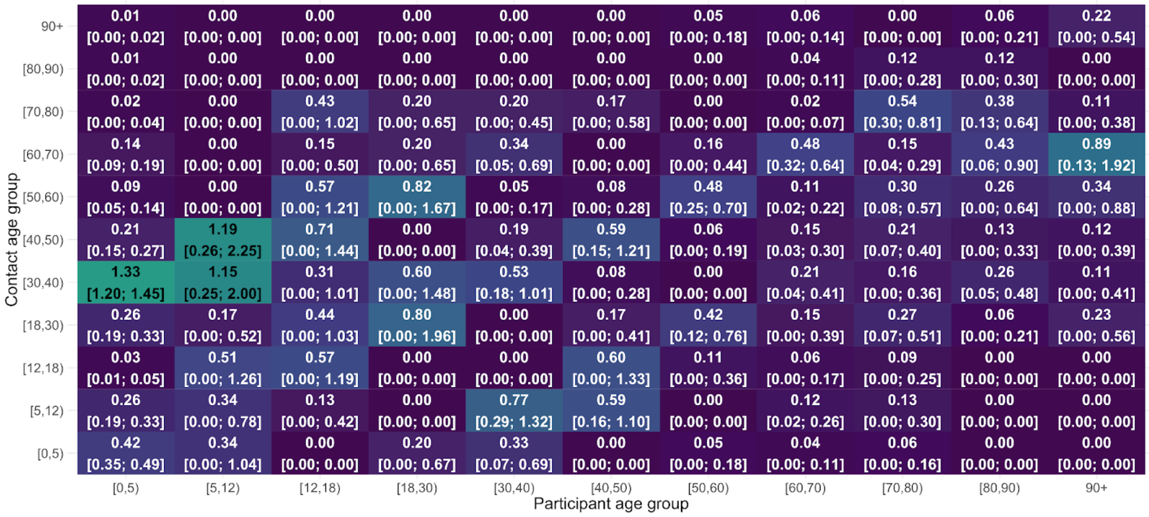

Figure S6: Social contact matrices for participants reporting no answer regarding chronic conditions representing contacts *(Top)* not at home, and *(Bottom)* at home.

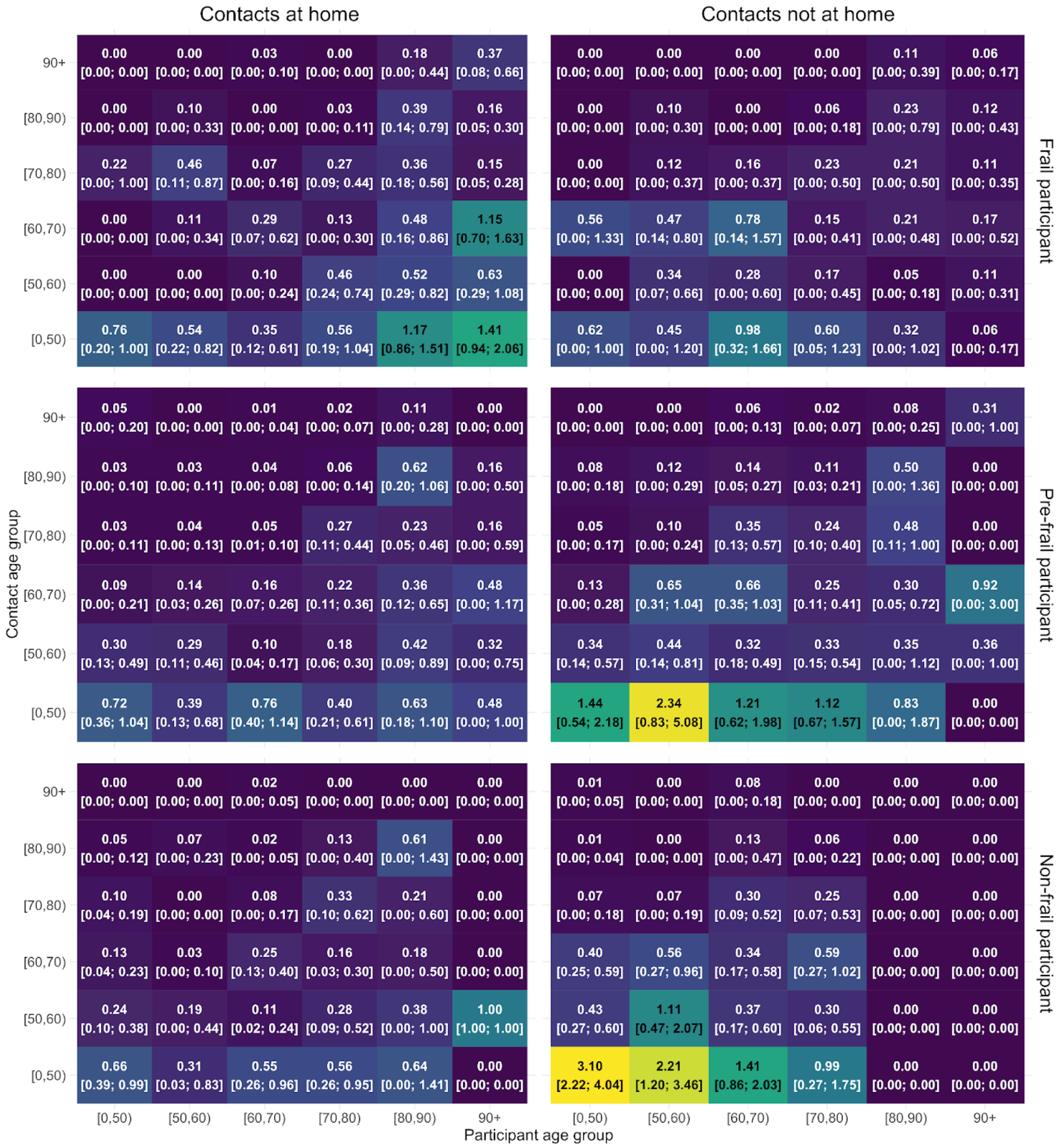

Figure S7. Contact matrices showing the mixing patterns of participants living in residential units alone based on their frailty level for contacts reported at and not at home with non-household members, together with 95% confidence intervals obtained from non-parametric bootstrap

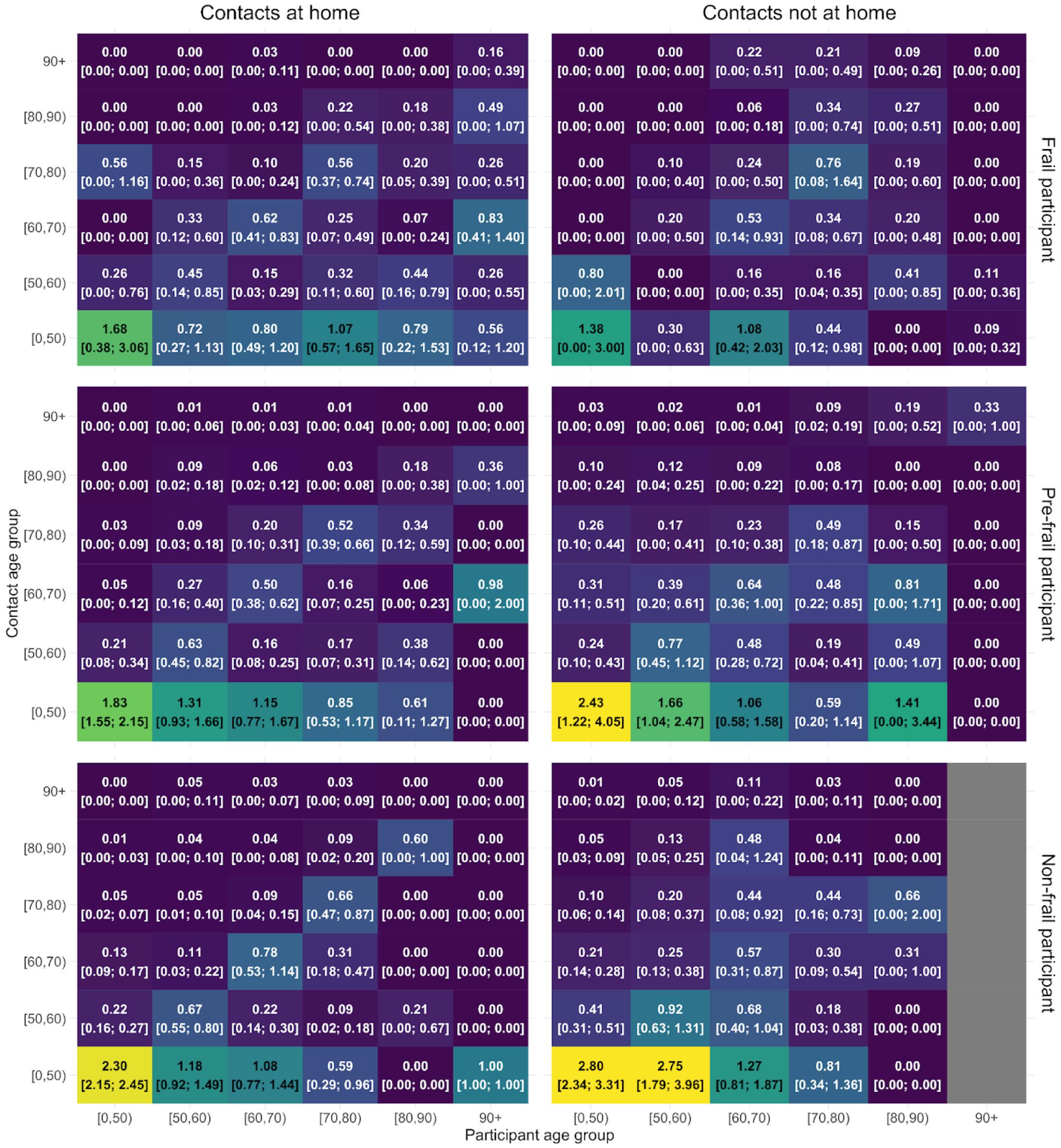

Figure S8. Contact matrices showing the mixing patterns of participants living with others in residential units based on their frailty level for contacts reported at and not at home with non-household members, together with 95% confidence intervals obtained from non-parametric bootstrap

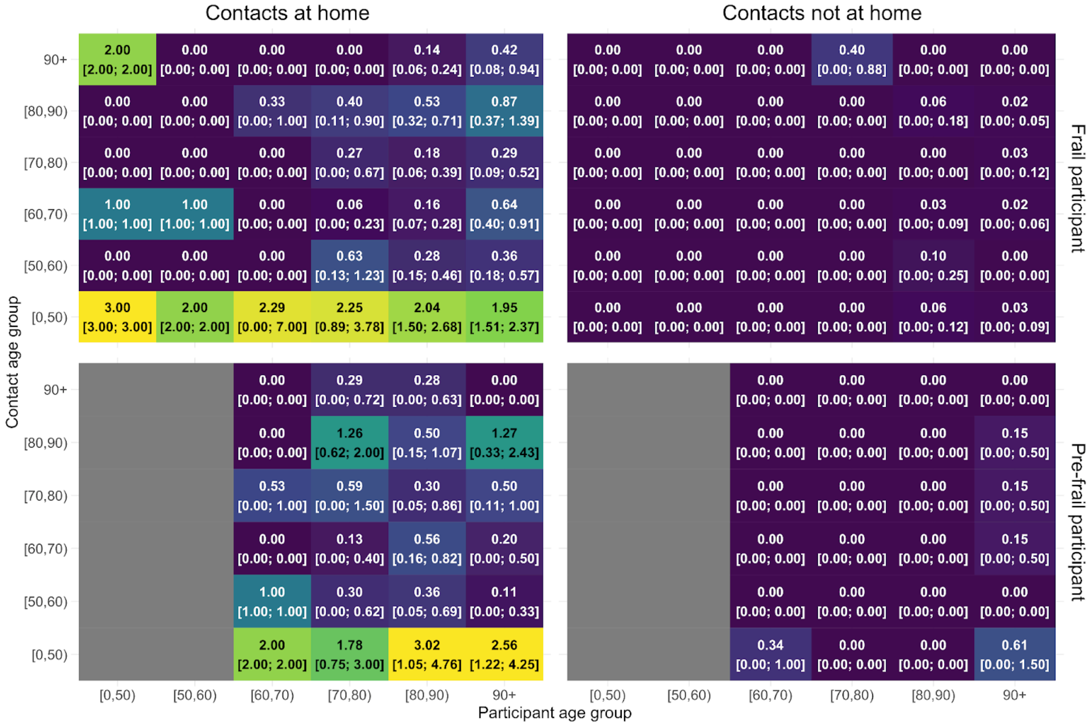

Figure S9. Contact matrices showing the mixing patterns of participants living in healthcare facilities based on their frailty level for contacts reported at and not at home with non-household members, together with 95% confidence intervals obtained from non-parametric bootstrap

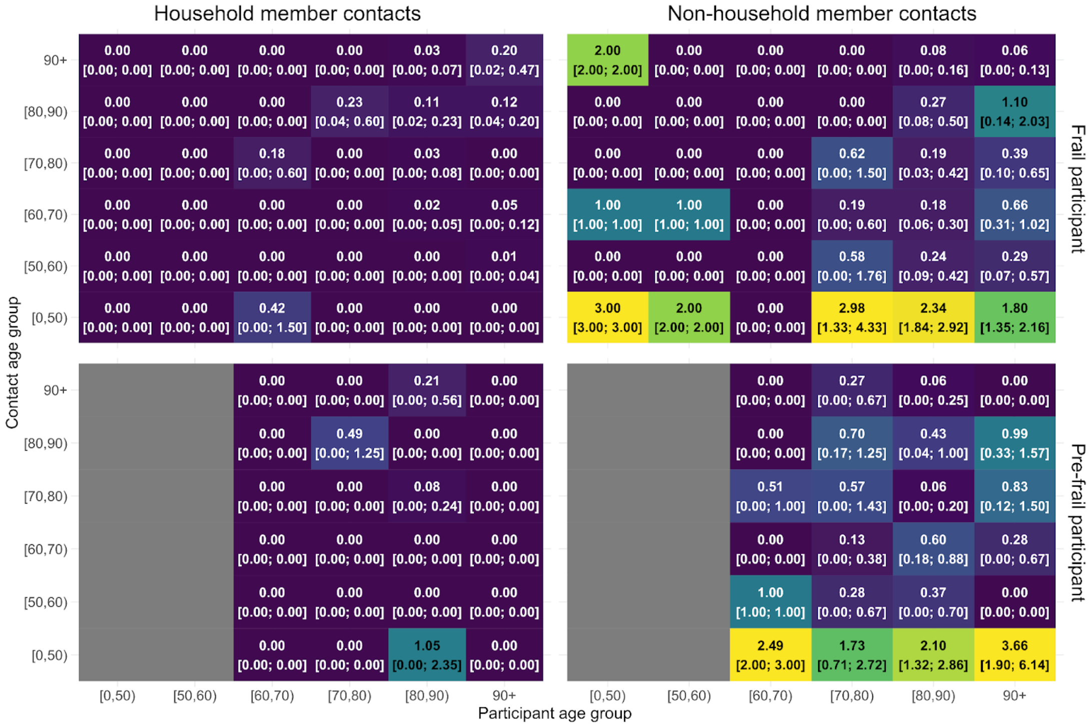

Figure S10. Contact matrices showing the mixing patterns of participants living in the healthcare facilities based on their frailty level for contacts reported with household and non-household members, together with 95% confidence intervals obtained from non-parametric bootstrap.

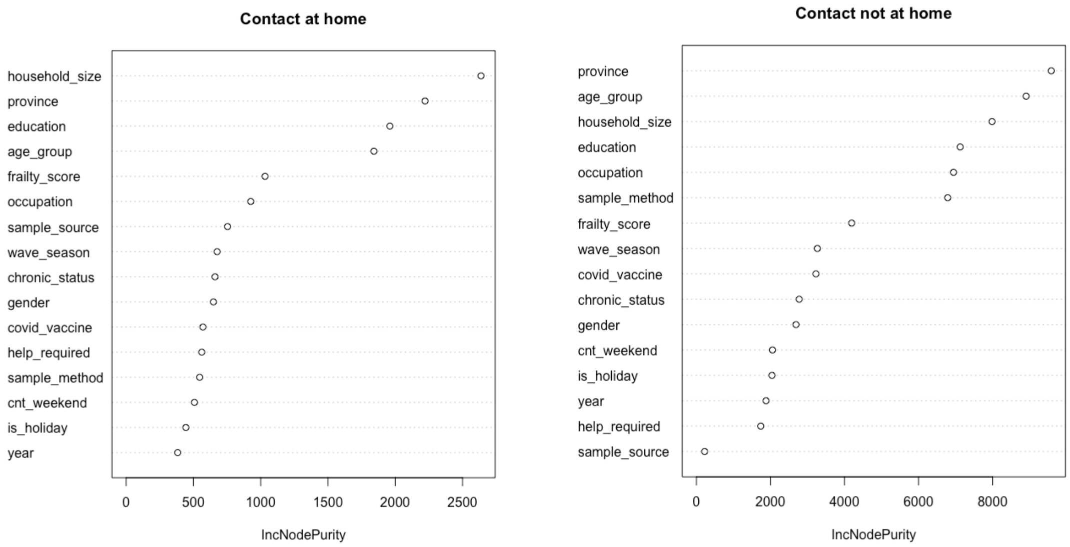

Figure S11: Variable importance calculated by the random forest

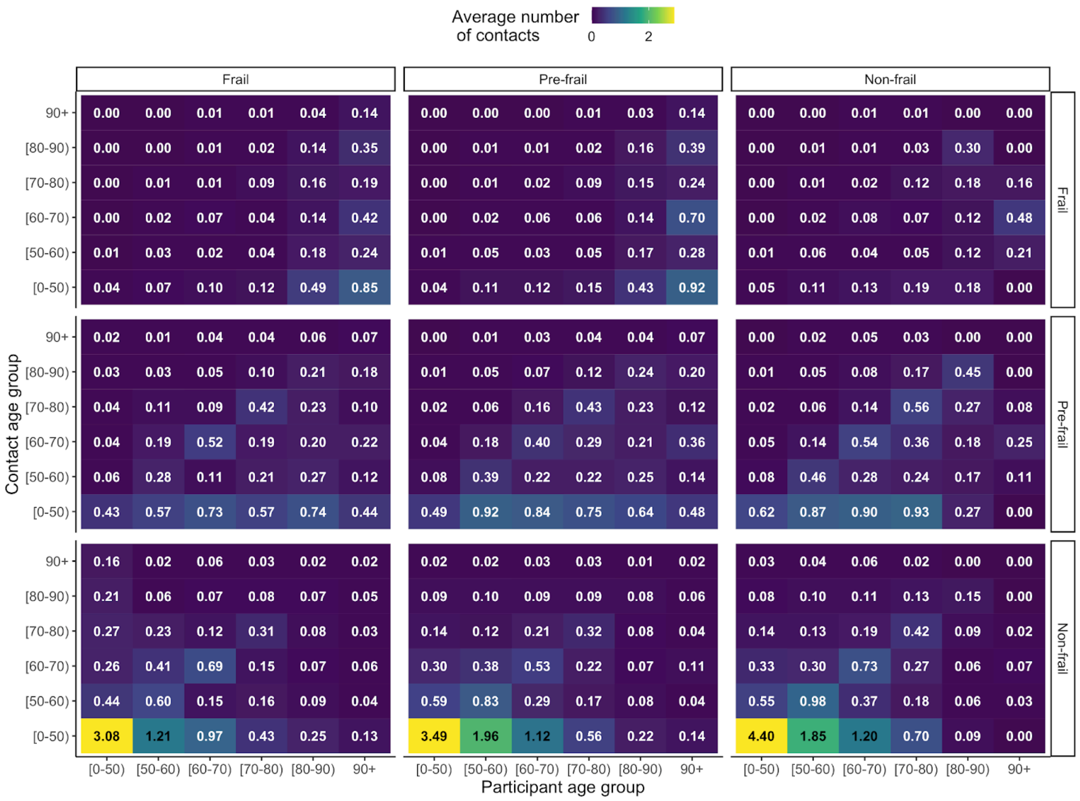

Figure S12. Decomposed contact matrices showing the mixing patterns between different frailty levels, assuming *proportionate* mixing between participants and contactees.

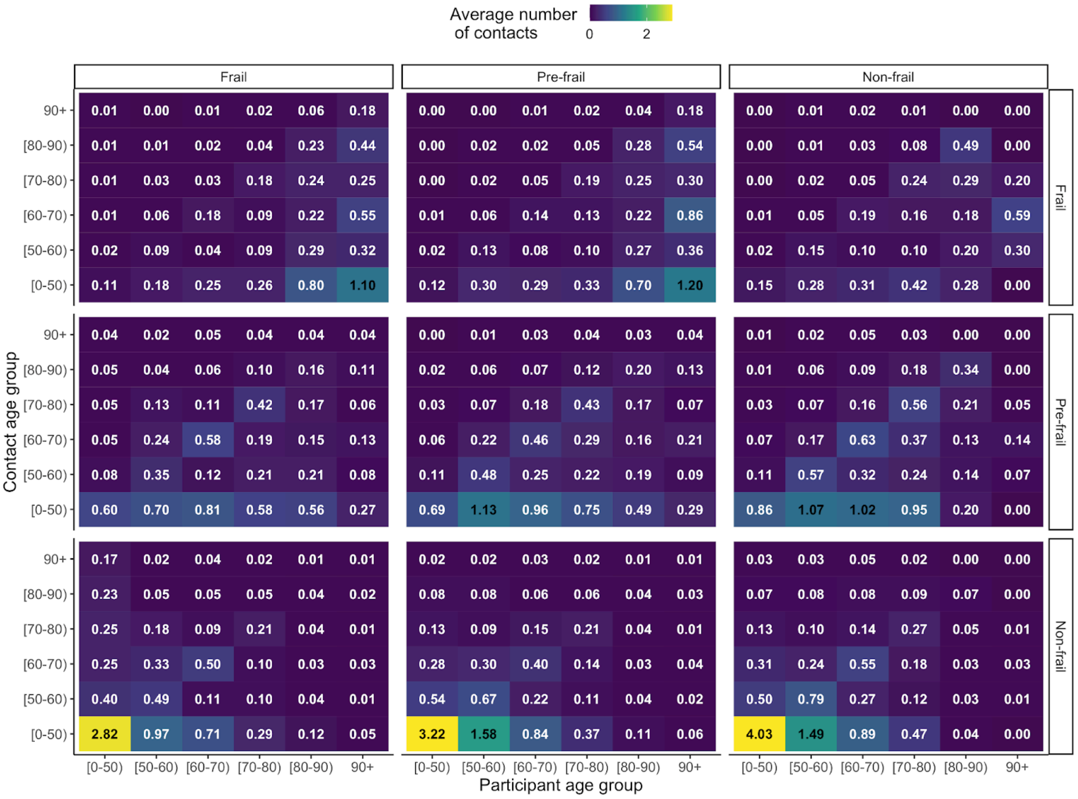

Figure S13. Decomposed contact matrices showing the mixing patterns between different frailty levels, assuming *uniform* mixing between participants and contactees.

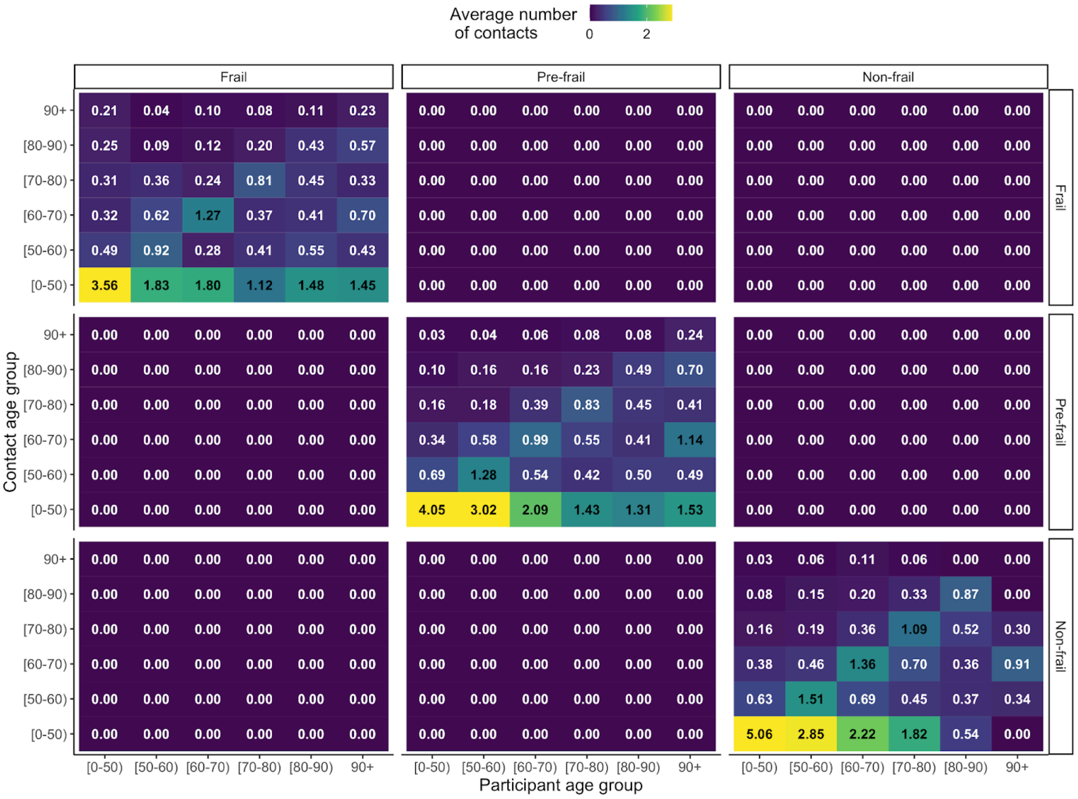

Figure S14. Decomposed contact matrices showing the mixing patterns between different frailty levels, assuming full *assortativity* mixing between participants and contactees.

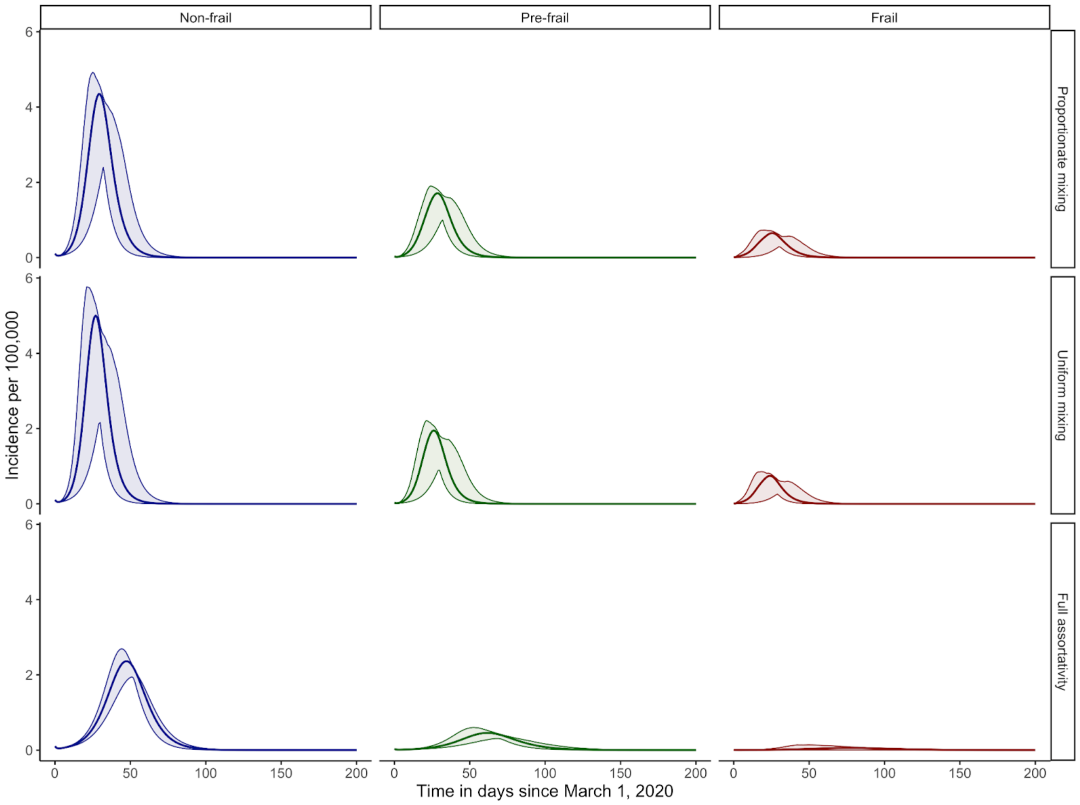

Figure S15. Comparison of epidemic curves together with 95% confidence intervals obtained from non-parametric bootstrap for the Belgian population with various frailty-based mixing patterns with the equal host- and disease-specific proportionality factor (q).

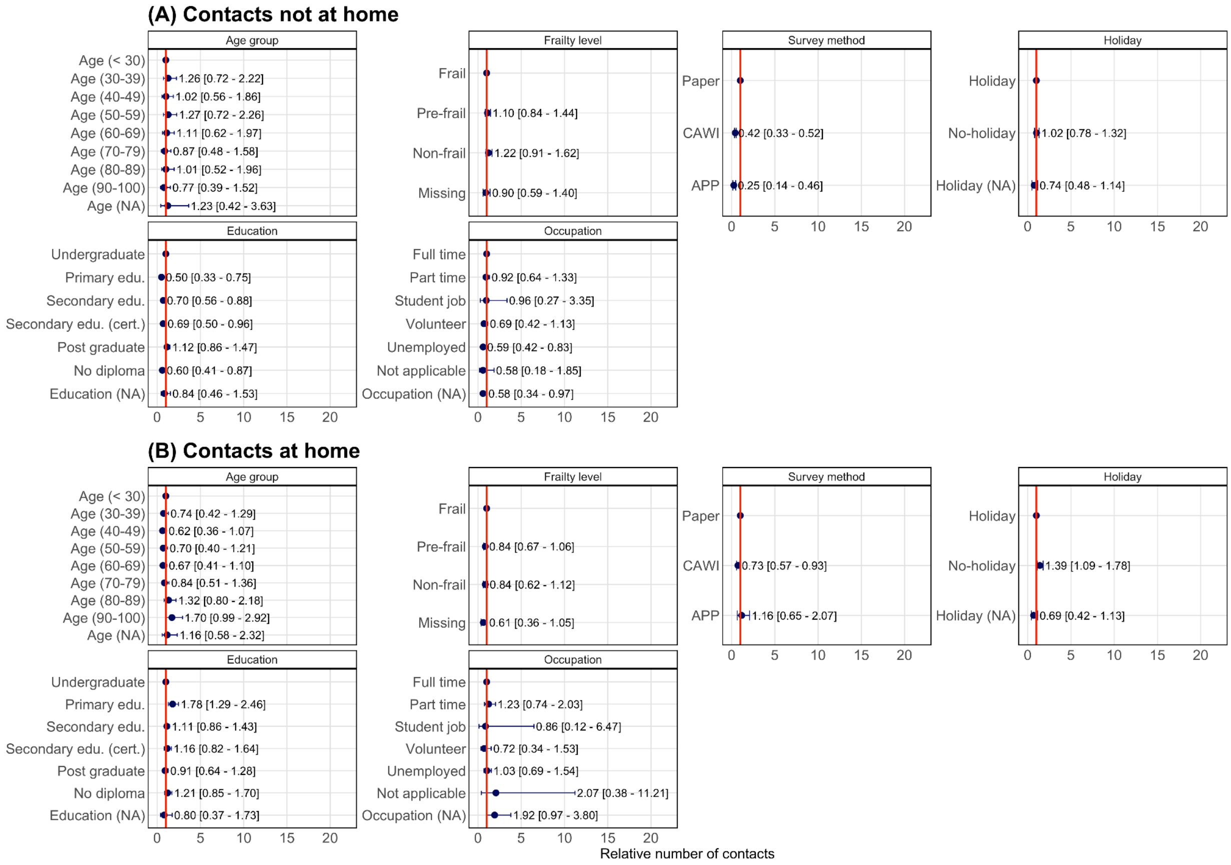

Figure S16. GAMLSS model for the reported number of contacts for those residing in a household of size 1.

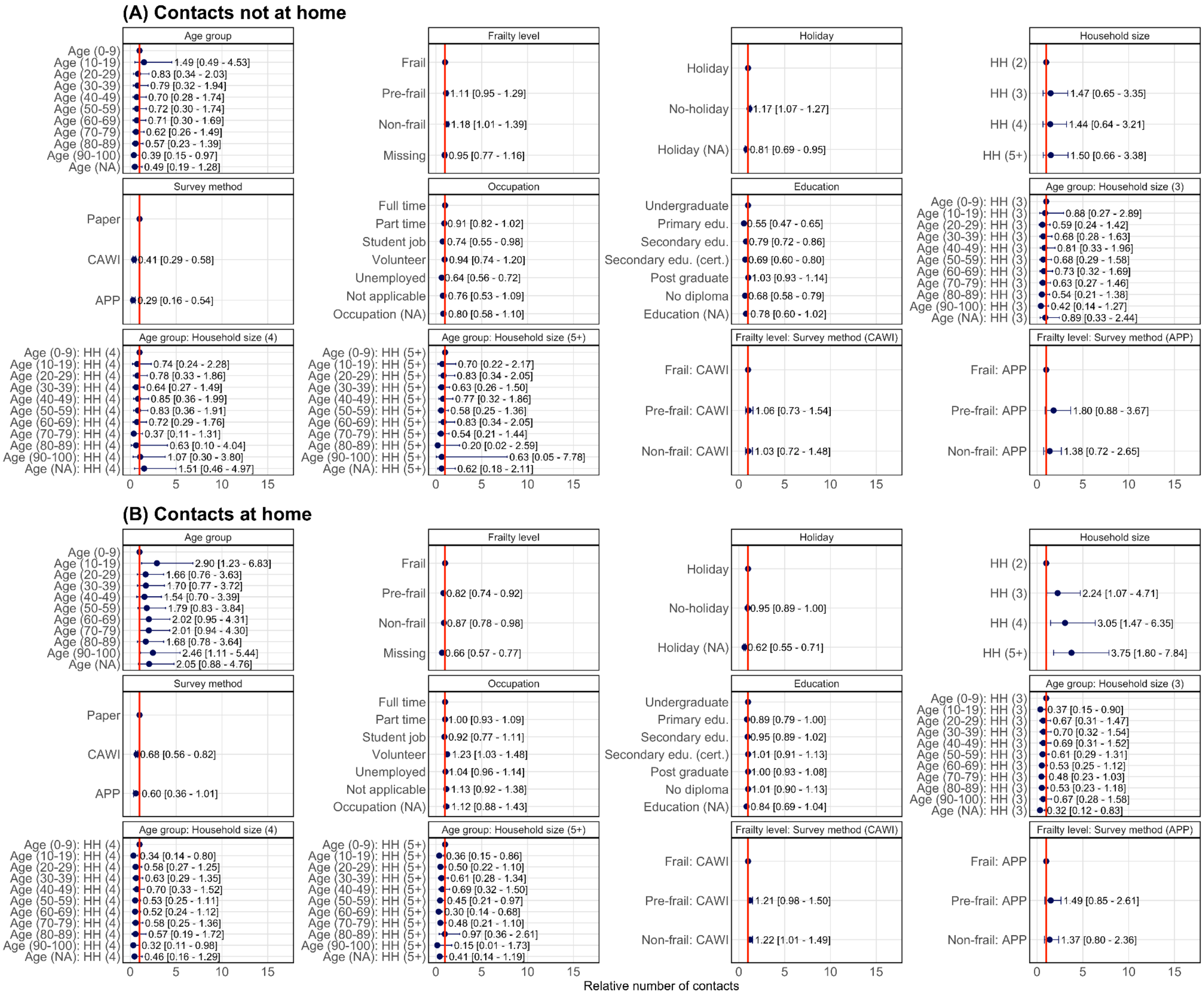

Figure S17. GAMLSS model for the reported number of contacts for those residing in a household of sizes 2, 3, 4, or 5+.

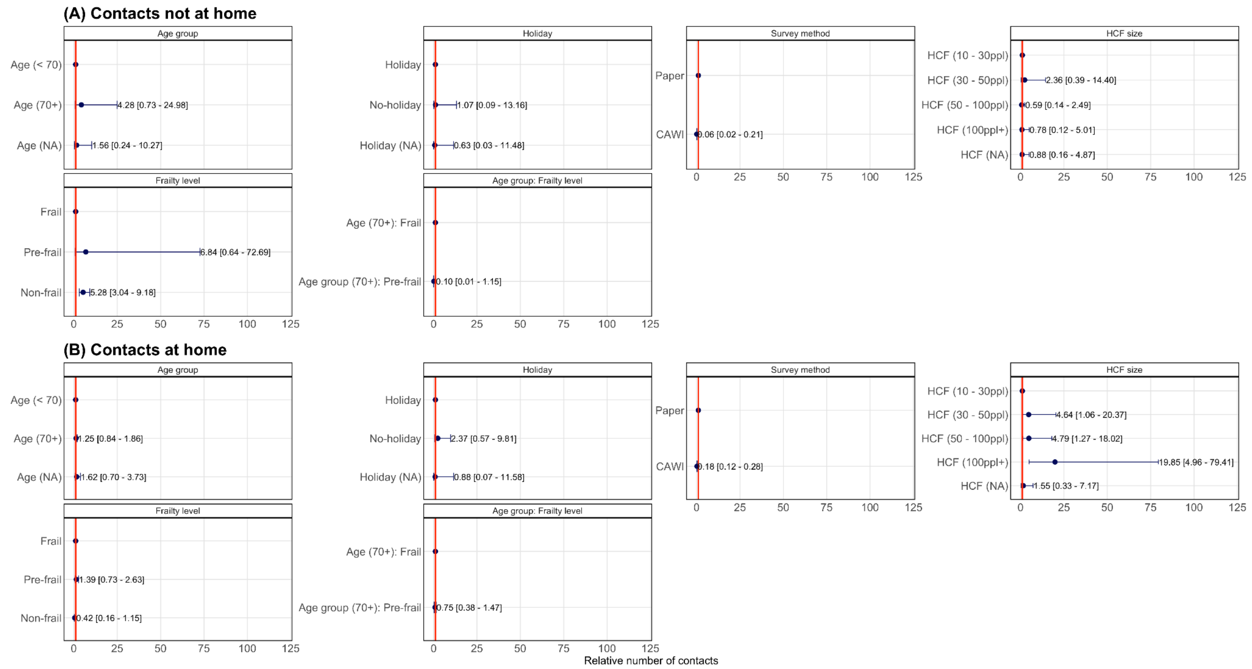

Figure S18. GAMLSS model for the reported number of contacts in healthcare facilities.

4. Supplementary materials (Tables)

Table S1: Overview of study populations, survey methods, and quota in the data collection.

Study population	**General population**	**Care facilities**	**Chronic conditions**	**ILI**	**App**
Survey methods	Paper/Online	Face-to-face interview	Paper/Online	Paper/Online	App
Recruitment	National Registry	List of facilities	National Registry	National Registry	National Registry
Age category: Target (Response ratio)	0-2: 228 (12%)<br>3-8: 150 (12%)<br>9-24: 304 (12%)<br>25-49: 475 (11%)<br>50-79: 750 (22%)<br>80-89: 130 (22%)<br>90-99: 60 (19%)	50-75: 150<br>75+: 120	50-79: 150 (11%)<br>80-89: 10 (10%)<br>90-99: 10 (11%)	27-75: 500	21-60: 160 (6%)

Quota (Total)	2097<br>(2529 + 1853)	270 (271)	170 (1160)	250 (16)	160 (166)
Age category: Total per age (% of the target achieved)	0-2: 325 (143%)<br>3-8: 177 (118%)<br>9-24: 373 (113%)<br>25-49: 458 (96%)<br>50-79: 933 (124%)<br>80-89: 185 (142%)<br>90-99: 78 (130%)<br><br>Boost sample:<br>21-99: 1853	50-75: 31 (21%)<br>75+: 238 (198%)<br><br>NA: 2	50-79: 1076 (717%)<br>80-89: 41 (410%)<br>90-99: 43 (430%)	26-75: 16 (3%)	21-60: 166 (104%)

Table S2: Parameters utilised for the discrete-time age-structured SEIR compartmental model.

**Notation**	**Description**	**Value**
γ	Average length of latency period	1.5 days
θ	Average length of pre-symptomatic infectious period	2.9 days
δ_1	Average length of infectious period when mildly infected (after pre-symptomatic phase)	2.4 days
δ_2	Average length of infectious period when mildly infected (after pre-symptomatic phase)	6.5 days
δ_3	Recovery period of hospitalised individuals' length of stay in the hospital	23.3 days
δ_4	Recovery period of individual length of stay in the ICU	23.2 days
q	Proportionality factor capturing contextual and host- and disease-specific characteristics	0.051
$\boldsymbol{\omega}$	The average time between symptoms onset and hospitalisation.	1.67 days
$\boldsymbol{\Phi}_1$	The proportion of symptomatic cases developing only mild symptoms	0.881
$\boldsymbol{p}$	The proportion of asymptomatic cases	0.129
$\boldsymbol{\mu}$	The probability of dying upon hospitalisation (Number of deaths relative to number of hospitalised individuals)	0.473

Table S3: Distribution of initial COVID-19 exposures (and population) across age groups (March 1, 2020) [1]

Age group	Incidence (population)	Age group	Incidence
0-50	11,716 (6,982,929)	70-80	372 (980,558)
50-60	1,119 (1,586,001)	80-90	264 (517,932)
60-70	466 (1,390,799)	90-100	101 (125,789)

Table S4: Summary statistics of the Epicurus study. Summary statistics for numeric variables are median (IQR) values and any unanswered questions (marked as NA or "No answer") in the survey were excluded from the table. Consequently, the percentages displayed in the table will not sum up to 100%.

	Wave 1 (N = 535)	Wave 2 (N = 1,285)	Wave 3 (N = 3,388)	No answer (N = 515)
**Survey date**	**2022-06-14 to 2023-06-26**	**2022-09-07 to 2023-01-20**	**2023-01-23 to 2023-05-09**	**-**
Unknown	0	0	0	515
**Sample source**				
Nat rep	466 (87%)	1,174 (91%)	3,298 (97%)	514 (100%)
Care facilities	69 (13%)	111 (8.6%)	90 (2.7%)	1 (0.2%)
**Sample method**				
Paper	310 (58%)	810 (63%)	2,090 (62%)	515 (100%)
CAWI	225 (42%)	467 (36%)	1,140 (34%)	0 (0%)
App	0 (0%)	8 (0.6%)	158 (4.7%)	0 (0%)
**Help required**				
Self answered	369 (69%)	971 (76%)	2,836 (84%)	307 (60%)
With help	159 (30%)	294 (23%)	486 (14%)	61 (12%)
No answer	7 (1.3%)	20 (1.6%)	66 (1.9%)	147 (29%)
**Education**				
Undergraduate degree	97 (18%)	291 (23%)	967 (29%)	51 (9.9%)
Diploma (primary education)	61 (11%)	139 (11%)	267 (7.9%)	61 (12%)
Diploma	115 (21%)	334 (26%)	937 (28%)	102 (20%)

(secondary education)				
Certificate (secondary education)	39 (7.3%)	92 (7.2%)	256 (7.6%)	46 (8.9%)
Postgraduate degree	61 (11%)	160 (12%)	532 (16%)	40 (5.8%)
No official diploma	152 (28%)	249 (19%)	378 (11%)	95 (18%)
No answer	10 (1.9%)	20 (1.6%)	51 (1.5%)	130 (2.5%)
**Province**				
Antwerpen	118 (22%)	367 (29%)	938 (28%)	95 (18%)
Limburg	80 (15%)	165 (13%)	517 (15%)	68 (13%)
Oost-Vlaanderen	135 (25%)	265 (21%)	695 (21%)	81 (16%)
West-Vlaanderen	106 (20%)	209 (16%)	574 (17%)	97 (19%)
Vlaams Brabant	82 (15%)	226 (18%)	544 (16%)	50 (9.7%)
Others	0 (0%)	8 (0.6%)	14 (0.4%)	3 (0.6%)
No answer	14 (2.6%)	45 (3.5%)	106 (3.1%)	121 (23%)
**Gender**				
Male	220 (41%)	597 (46%)	1,503 (44%)	208 (40%)
Female	311 (58%)	682 (53%)	1,858 (55%)	209 (41%)
Others	0 (0%)	0 (0%)	2 (< 0.1%)	0 (0%)
No answer	4 (0.7%)	6 (0.5%)	25 (0.7%)	98 (19%)
**Occupation**				
Full time	122 (23%)	340 (26%)	1,011 (30%)	64 (12%)
Part time	37 (6.9%)	115 (8.9%)	416 (12%)	26 (5%)
Student job	15 (2.8%)	23 (1.8%)	49 (1.4%)	4 (0.8%)
Volunteer	7 (1.3%)	25 (1.9%)	83 (2.4%)	10 (1.9%)
Unemployed	221 (41%)	560 (44%)	1,524 (45%)	274 (53%)
Not applicable	128 (24%)	201 (16%)	263 (7.8%)	24 (4.7%)
No answer	5 (0.9%)	21 (1.6%)	42 (1.2%)	113 (22%)

**Age group**				
0-9	109 (20%)	154 (12%)	174 (5.1%)	16 (3.1%)
10-19	31 (5.8%)	73 (5.7%)	132 (3.9%)	16 (3.1%)
20-29	39 (7.3%)	74 (5.8%)	212 (6.3%)	8 (1.6%)
30-39	43 (8%)	93 (7.2%)	315 (9.3%)	16 (3.1%)
40-49	40 (7.5%)	93 (7.2%)	302 (8.9%)	20 (3.9%)
50-59	42 (7.9%)	190 (15%)	562 (17%)	38 (7.4%)
60-69	68 (13%)	242 (19%)	818 (24%)	95 (18%)
70-79	60 (11%)	181 (14%)	610 (18%)	112 (22%)
80-90	59 (11%)	108 (8.4%)	120 (3.5%)	47 (9.1%)
90-100	39 (7.3%)	71 (5.5%)	102 (3.0%)	30 (5.8%)
No answer	5 (0.9%)	6 (0.5%)	41 (1.2%)	117 (23%)
**Chronic status**				
Chronic condition	155 (29%)	440 (34%)	1,134 (33%)	183 (36%)
No chronic condition	285 (53%)	730 (57%)	2,024 (60%)	162 (31%)
No answer	95 (18%)	115 (8.9%)	230 (6.8%)	170 (33%)
**Resident status**				
Residential unit	311 (58%)	925 (72%)	2,852 (84%)	362 (70%)
With parents	137 (26%)	218 (17%)	373 (11%)	28 (5.4%)
Family other than parents	4 (0.7%)	7 (0.5%)	15 (0.4%)	0 (0%)
ROB	19 (3.6%)	24 (1.9%)	43 (1.3%)	1 (0.2%)
RVT	27 (5%)	64 (5%)	19 (0.6%)	1 (0.2%)
Others	27 (5%)	24 (1.9%)	31 (0.9%)	4 (0.8%)
No answer	10 (1.9%)	23 (1.8%)	55 (1.6%)	119 (23%)
**Household size**				
1	42 (7.9%)	124 (9.6%)	392 (12%)	54 (10%)
2	137 (26%)	399 (31%)	1,224 (36%)	139 (27%)
3	83 (16%)	216 (17%)	675 (20%)	76 (15%)

4	129 (24%)	232 (18%)	540 (16%)	28 (5.4%)
5+	53 (9.9%)	158 (12%)	317 (9.4%)	37 (7.2%)
ROB/RVT	46 (8.6%)	88 (6.8%)	62 (1.8%)	2 (0.4%)
No answer	45 (8.4%)	68 (5.3%)	178 (5.3%)	179 (35%)
**Holiday**				
Yes	279 (52%)	122 (9.5%)	541 (16%)	0 (0%)
No	256 (48%)	1,163 (91%)	2,847 (84%)	0 (0%)
No answer	0 (0%)	0 (0%)	0 (0%)	515 (100%)
**Year**				
2022	360 (67%)	1,251 (97%)	0 (0%)	0 (0%)
2023	175 (33%)	34 (2.6%)	3,388 (100%)	0 (0%)
No answer	0 (0%)	0 (0%)	0 (0%)	515 (100%)
**Frailty score**				
Frail	90 (17%)	204 (16%)	448 (13%)	88 (17%)
Pre-frail	129 (24%)	366 (28%)	1,153 (34%)	101 (20%)
Non-frail	293 (55%)	651 (51%)	1,617 (48%)	120 (23%)
Missing	23 (4.3%)	64 (5%)	170 (5%)	206 (40%)

Table S5. Summary of the preferred survey method for the participants, stratified by age group

**Age group**	**Paper**	**CAWI**	**App**	**Age group**	**Paper**	**CAWI**	**App**
**0-9**	208 (45.92%)	245 (54.08%)	-	**60-69**	921 (75.31%)	299 (24.45%)	3 (0.25%)
**10-19**	138 (54.76%)	114 (45.24%)	-	**70-79**	814 (84.53%)	149 (15.47%)	-
**20-29**	127 (38.14%)	154 (46.25%)	52 (15.62%)	**80-89**	272 (81.44%)	62 (18.56%)	-
**30-39**	184 (39.40%)	237 (50.75%)	46 (9.85%)	**90-100**	193 (79.75%)	49 (20.25%)	-

**40-49**	216 (47.47%)	208 (45.71%)	31 (6.81%)	**No answer**	169 (100%)	-	-
**50-59**	483 (58.05%)	315 (37.86%)	34 (4.09%)				

Table S6: Summary statistics of the reported contacts (Median (IQR))

	**Wave 1 (N = 3,110)**	**Wave 2 (N = 7,218)**	**Wave 3 (N = 19, 392)**	**No answer (N = 1,655)**
**Contact date**<br><br>Unknown	**2022-06-13 to 2023-06-25**<br><br>**0**	**2022-09-06 to 2023-01-19**<br><br>**0**	**2023-01-22 to 2023-05-08**<br><br>**0**	**-**<br><br>**1,655**
**Type of day (contact)**				
Weekday	2,475 (80%)	5,670 (79%)	15,312 (79%)	0 (0%)
Weekend	645 (20%)	1,548 (21%)	4,080 (21%)	0 (0%)
No answer	0 (0%)	0 (0%)	0 (0%)	1,655 (100%)
**Contact at home**	1,393 (45%)	3,043 (42%)	7,348 (38%)	522 (32%)
**Physical contact**				
Yes	1,806 (58%)	3,799 (53%)	9,121 (47%)	644 (39%)
No	918 (30%)	3,001 (42%)	7,742 (40%)	401 (24%)
No answer	386 (12%)	418 (5.8%)	2,529 (13%)	610 (37%)
**Contact duration**				
≤ 5 minutes	152 (4.9%)	372 (5.2%)	954 (4.9%)	66 (40%)
5 - 15 minutes	371 (12%)	763 (11%)	1,815 (9.4%)	90 (5.4%)
15 min - 1 hour	389 (13%)	1,277 (18%)	3,244 (17%)	190 (11%)
1 - 4 hours	741 (24%)	2,041 (28%)	5,620 (29%)	360 (22%)
≥ 4 hours	1,068 (34%)	2,416 (33%)	5,437 (28%)	438 (26%)
No answer	389 (13%)	349 (4,8%)	2,322 (12%)	521 (31%)
**Number of contacts**	5 (2, 7)	4 (2, 7)	5 (3, 7)	1 (0, 4)

Table S7: The likelihood ratio test for GAMLSS, p-value <0.05 indicates that the variable is retained or added to the model. If a variable is significant in either model (at home or not at home), it will be used in both GAMLSS models.

	**p-value**	
	**Contacts at home**	**Contacts not at home**
Sample source	< 0.05	0.247
Help required	< 0.05	0.256
Chronic status	0.445	0.147
Covid-19 vaccination status	0.977	0.139
Education	0.982	< 0.05
Province	0.193	0.401
Age group: Frailty score	0.348	0.477
Holiday: Contact day	0.851	< 0.05
Age group: Contact day	0.712	0.249
Age group: Holiday	0.133	< 0.05
Frailty score: Help required	< 0.05	0.487

Table S8: NBI Generalised Linear Model Summary Statistics (95% CI) for Outside- and Inside- home contacts. The asterisk (*) indicates significance of the variable (p-value < 0.05)

Covariates	RI (Outside)	RI (Inside)
**Sample source**		
General		
Care facilities	0.905 [0.681; 1.202]	2.141* [1.797; 2.550]
**Help required**		
Self answered		
With help	0.825 [0.662; 1.028]	1.497* [1.325; 1.692]
No answer	1.229 [0.723; 2.087]	0.929 [0.623; 1.386]
**Age group**		
0-9	-	-
10-19	1.345 [0.905; 1.999]	1.258* [1.033; 1.532]

20-29	0.960 [0.596; 1.547]	1.089 [0.827; 1.435]
30-39	0.855 [0.539; 1.357]	1.063 [0.799; 1.413]
40-49	0.605* [0.373; 0.981]	1.152 [0.870; 1.527]
50-59	0.679 [0.433; 1.063]	1.065 [0.816; 1.390]
60-69	0.671 [0.434; 1.036]	1.173 [0.907; 1.518]
70-79	0.616* [0.395; 0.962]	1.210 [0.928; 1.577]
80-89	0.613 [0.351; 1.072]	1.163 [0.820; 1.649]
90-100	0.599 [0.330; 1.085]	0.847 [0.549; 1.309]
No answer	0.944 [0.458; 1.947]	1.174 [0.662; 2.083]
**Gender**		
Male	-	-
Female	1.077* [1.012; 1.146]	1.066* [1.020; 1.113]
Others	1.074 [0.278; 4.144]	2.676* [1.202; 5.956]
No answer	0.680 [0.456; 1.012]	1.153 [0.862; 1.541]
**Household size**		
1	-	-
2	0.854* [0.770; 0.948]	1.233* [1.111; 1.367]
3	0.859* [0.764; 0.964]	1.562* [1.403; 1.740]
4	0.882 [0.776; 1.003]	2.009* [1.799; 2.242]
5+	0.866* [0.753; 0.995]	2.315* [2.060; 2.602]
ROB/RVT	0.733 [0.529; 1.014]	1.249* [1.034; 1.510]
No answer	0.835* [0.718; 0.972]	1.269* [1.103; 1.460]
**Survey method**		
Paper	-	-
CAWI	0.436* [0.406; 0.468]	0.862* [0.823; 0.904]
App	0.404* [0.337; 0.485]	0.886 [0.782; 1.005]
**Occupation**		
Full time	-	-
Part time	0.914 [0.824; 1.014]	0.987 [0.913; 1.066]

Student job	0.719* [0.543; 0.950]	0.927 [0.780; 1.102]
Volunteer	1.010 [0.817; 1.250]	1.194* [1.012; 1.409]
Unemployed	0.641* [0.573; 0.716]	1.087* [1.001; 1.180]
Not applicable	0.800 [0.565; 1.133]	1.158 [0.953; 1.407]
Not answer	0.829 [0.636; 1.080]	1.219 [0.987; 1.507]
**Frailty score**		
Frail	-	-
Pre-frail	1.095 [0.963; 1.246]	0.992 [0.906; 1.086]
Non-frail	1.175* [1.029; 1.342]	1.023 [0.932; 1.123]
Missing	0.900 [0.751; 1.077]	0.899 [0.777; 1.041]
**Education**		
Undergraduate degree	-	-
Diploma (primary education)	0.599* [0.513; 0.699]	0.978 [0.888; 1.078]
Diploma (secondary education)	0.778* [0.717; 0.843]	1.002 [0.942; 1.065]
Certificate (secondary education)	0.709* [0.617; 0.814]	0.981 [0.890; 1.081]
Postgraduate degree	1.052 [0.959; 1.154]	1.005 [0.937; 1.078]
No official diploma	0.703* [0.603; 0.821]	1.021 [0.922; 1.131]
No answer	0.724* [0.571; 0.918]	0.948 [0.759; 1.185]
**Year: Wave**		
2022: Wave 1 (Summer)	0.878 [0.771; 1.000]	0.930 [0.849; 1.017]
2023: Wave 1 (Summer)	1.154 [0.957; 1.391]	1.097 [0.986; 1.220]
2022: Wave 2 (Fall)	0.870* [0.808; 0.937]	0.993 [0.944; 1.045]
2023: Wave 2 (Fall)	0.850 [0.586; 1.232]	0.941 [0.732; 1.209]
2023: Wave 3 (Winter)	-	-
**Holiday: Day of contact**		
Holiday (No): Weekend	-	-
Holiday (No): Weekday	1.319* [1.218; 1.427]	0.854* [0.810; 0.901]
Holiday (Yes): Weekend	0.791 [0.592; 1.057]	0.963 [0.817; 1.135]
Holiday (Yes): Weekday	0.780 [0.606; 1.005]	0.818* [0.709; 0.943]

Age group: Holiday		
0-9: Holiday (No)	-	-
10-19: Holiday (No)	0.801 [0.531; 1.209]	0.749* [0.608; 0.922]
20-29: Holiday (No)	0.577* [0.394; 0.847]	0.874 [0.696; 1.098]
30-39: Holiday (No)	0.574* [0.409; 0.804]	1.004 [0.800; 1.260]
40-49: Holiday (No)	0.907 [0.626; 1.314]	0.916 [0.736; 1.142]
50-59: Holiday (No)	0.729* [0.535; 0.995]	0.905 [0.743; 1.103]
60-69: Holiday (No)	0.725* [0.541; 0.971]	0.849 [0.702; 1.026]
70-79: Holiday (No)	0.662* [0.485; 0.904]	0.819* [0.670; 1.000]
80-89: Holiday (No)	0.554* [0.345; 0.888]	0.769 [0.564; 1.048]
90-100: Holiday (No)	0.495* [0.292; 0.839]	1.352 [0.902; 2.027]
No answer: Holiday (No)	0.503 [0.251; 1.007]	0.843 [0.467; 1.519]
**Frailty score: Help required**		
Non-frail: With help	1.095 [0.822; 1.458]	0.685* [0.584; 0.803]
Pre-frail: With help	1.218 [0.907; 1.635]	0.779* [0.654; 0.927]
Missing: With help	1.005 [0.584; 1.728]	0.739 [0.508; 1.075]
Frail: Self answered	-	-
Non-frail: No answer	0.846 [0.458; 1.563]	1.324 [0.840; 2.087]
Pre-frail: No answer	0.588 [0.308; 1.121]	0.903 [0.525; 1.551]
Missing: No answer	0.669 [0.319; 1.405]	0.841 [0.413; 1.714]

References

1. Willem L *et al.* 2024 The impact of quality-adjusted life years on evaluating COVID-19 mitigation strategies: lessons from age-specific vaccination roll-out and variants of concern in Belgium (2020-2022). *BMC Public Health* **24**, 1171. (doi:10.1186/s12889-024-18576-w)
